# Relative contributions of public and domestic transmission domains in cholera outbreaks in displacement camps: an exploratory agent-based modeling study

**DOI:** 10.1017/S0950268826101575

**Published:** 2026-05-13

**Authors:** Tarek Jaber, Eline Boelee, Peter Kjær Mackie Jensen, Sake J. De Vlas, Bartel Van de Walle

**Affiliations:** 1 UNU-MERIT, Maastricht, Netherlands; 2 Maastricht University, Maastricht, Netherlands; 3 Deltares, Utrecht, Netherlands; 4 Institute for Risk Assessment Sciences (IRAS), Utrecht University, Utrecht, Netherlands; 5 Department of Public Health, Global Health Section, University of Copenhagen, Copenhagen, Denmark; 6 Department of Public Health, Erasmus MC, University Medical Center Rotterdam, Rotterdam, Netherlands

**Keywords:** computational modeling, epidemics, internally displaced persons, NetLogo, refugees

## Abstract

Cholera is associated with devastating outbreaks among forcibly displaced people. Insights into the relative contributions of the public (extra-household) and domestic (intra-household) domains to cholera spread in camps, as well as the circumstances under which each may drive transmission, can support the design of response strategies. However, these have yet to be systematically investigated. We developed an agent-based model of cholera transmission in camps informed by a rapid appraisal conducted in Northeast Nigeria, an expert consultation, and humanitarian minimum standards. We simulated outbreaks in a stylized camp that meets water quantity standards and compared this with conditions where water supply is overwhelmed or compromised following floods and population influxes. We found that domestic transmission can exclusively drive cholera outbreaks. However, unless hygiene conditions are extremely poor and water is not adequately chlorinated, these outbreaks appear to be small. Following shocks, outbreaks can be large and progress rapidly. Although they are initially shaped by the public domain, domestic domain transmission can sustain or exacerbate them. We recommend directing humanitarian and development activities towards mitigating the consequences of extreme weather events and unplanned population influxes, as well as developing adaptive preparedness and response strategies that explicitly and comprehensively address them.

## Introduction

Cholera is an acute diarrheal disease caused by toxigenic strains of *Vibrio cholerae* bacteria. Half of internally displaced persons (IDPs) and around 20% of cross-border refugees reside in displacement camps [1]. These camps are often characterized by limited access to sufficient clean water and adequate sanitation facilities. As a consequence, forcibly displaced people may be exposed to enteric pathogens, including *V. cholerae* [2,3,4,5], one of the leading causes of infectious disease outbreaks in camps [6,7].

Many interdependent factors may contribute to and worsen outbreaks of *V. cholerae* in camps. These include inadequate access to water that is safe for drinking and sufficient to maintain good domestic and personal hygiene, limited healthcare capacity, overcrowding, open defecation, poor adherence to hand hygiene, and unsafe water and food storage and handling practices [4]. In light of improved cholera preparedness, multisectoral coordination, and adherence to minimum humanitarian standards, cholera outbreaks in camps have decreased in severity [8]. Large outbreaks may still occur when preparedness measures are jeopardized, for example following extreme weather events or population influxes that overwhelm or compromise water, sanitation, and hygiene (WaSH) facilities at country level [9,10,11,12], but particularly in displacement camps [4,8,13]. These key drivers were identified through scoping literature reviews of outbreaks of diarrheal and fecal-orally transmitted diseases in displacement camps [4,13]. In response, public health measures ought to target all exposure routes to *V. cholerae*. Nevertheless, under these circumstances and in resource-constrained settings, it might be necessary to prioritize interventions and delivery strategies, requiring a thorough understanding of cholera transmission dynamics. These, however, are highly contextual [14] and poorly understood in humanitarian crises [15].

Fecal-oral transmission of *V. cholerae* can be conceptualized as occurring in the domestic (i.e., intra-household) or the public (i.e., extra-household) domain [16]. Accordingly, exposure to *V. cholerae* may occur through an environmental point source (e.g., contaminated surface water), or alternatively, the consumption of water or food contaminated at the household level. Insights into the relative contributions of the two domains, as well as the conditions under which each may drive cholera transmission, can inform preparedness and response strategies and help identify the merits of targeted interventions in displacement camps. While past investigations indicate that both domains of transmission may play a role in outbreaks of *V. cholerae* in camps [17,18,19,20,21], the contributions of each may not be inferred from case–control studies alone [22].

Many epidemiological models have been developed to better understand cholera transmission dynamics. For instance, a compartmental model formulated by Codeço [23] illustrated the effects of climatic events in shaping cholera transmission through their impact on the rates of contact with, and contamination of, water sources. Hartley et al. [24] further incorporated the role of a transient, more infectious state of freshly shed *V. cholerae* in reproducing the dynamics of cholera outbreaks. They suggested that outbreaks may be simultaneously shaped by two transmission pathways: isolated cases emerge from exposure to an environmental reservoir of *V. cholerae*, followed by clusters of cases resulting from local fecal-oral transmission and exposure to freshly shed *V. cholerae*. Agent-based models (ABMs) have also been used to explore cholera spread [25,26]. For example, Crooks and Hailegiorgis [26] developed an ABM of cholera spread in the Dadaab refugee camp in Kenya, simulating outbreaks as emergent phenomena following either the contamination of a borehole or the spread of the pathogen through surface water run-off. Such models explicitly simulate the behavior of individual agents that interact with each other and their environment [27], and may incorporate spatial structure, social networks, and heterogeneity in individual behaviors and characteristics. Accordingly, ABMs can be used to simulate water collection behavior in displacement camps, as well as fecal contamination of household-level containers and local transmission of *V. cholerae*. They can also account for residual chlorine decay. Water in displacement camps is often chlorinated to levels between 0.2 and 0.5 mg/L and distributed through tap stands [28]. The free residual chlorine (FRC) in treated water protects against post-collection microbiological contamination. However, once water is collected and stored in household-level containers, FRC decays over time [29]. Absent residual protection, water in household-level containers may be contaminated and consequently contribute to the spread of *V. cholerae*.

This study uses an ABM to investigate cholera transmission dynamics in a camp setting, focusing on distinguishing between public and domestic domains. Specifically, we quantify how the relative contribution of these domains changes over time under (i) a baseline scenario and (ii) shock scenarios such as a rapid population influx and heavy rainfall and subsequent flooding. By systematically varying key WaSH-related parameters (e.g., access to safe water, chlorine residual, or hygiene-related behaviors), we identify conditions under which outbreaks are primarily shaped and driven by public or domestic transmission. The results are intended to inform where and when WaSH interventions are likely to have the greatest impact during both stable periods and shocks.

## Methods

### Model description

The ABM is implemented in NetLogo [30], a modeling environment for agent-based simulation. A complete, detailed model description, following the ODD (Overview, Design concepts, Details) protocol [31,32] is provided in Appendix A, while some of the model parameters are discussed here and presented in the table below.

Our ABM is used to describe how acute population influxes and flood events may affect the percentage of infections emanating from both public and domestic transmission domains, as well as the size and timescale of cholera outbreaks. The model includes the following entities: PoCs, shelters, water facilities, and health facilities. PoCs are persons of concern to the United Nations High Commissioner for Refugees (UNHCR). These include refugees, asylum-seekers, and IDPs. Camps are organized into modular planning units, among which are communities and blocks [33]. Based on UNHCR’s principles and standards for settlement planning [33], blocks in the ABM consist of 16 communities, composed of 16 shelters each. Shelters are aggregations of PoCs. Each block includes a water facility entity, representing five taps for 1,250 people [28]. These deliver water throughout the day, with the FRC level set by a global parameter. There is one health facility in the camp. State variables characterizing these entities are presented in Appendix A.

The model is 150 × 150 patches, the environment representing 900,000 m^2^. Every patch represents 40 m^2^ to allow for 5.5 m^2^ of covered living area per person [33], for a household of six. Every time step represents 1 h to capture residual chlorine decay [29], and to allow for multiple water collection trips in a day. Simulations are run for a duration of 90 days or until outbreak termination, i.e., two consecutive weeks without new infections [34].

The processes that are repeated every time step between 06:00 and 22:00 for all simulation experiments are: (1) Shelters update FRC in household-level containers based on chlorine decay parameters empirically derived from data collected in refugee camps in South Sudan [29]. These parameters captured the combined effect of all factors that may have contributed to chlorine decay, including ambient temperature and water handling practices. The parameters for South Sudan were used to represent a worst-case scenario of rapid chlorine decay; (2) PoCs update their health status. PoCs can be susceptible, exposed (i.e., consumed water contaminated with *V. cholerae*, but not yet infectious), infected, or recovered; (3) PoCs are prompted to collect water if the quantity stored in household-level containers is less than or equal to the maximum amount that may be consumed in one time step. They choose among multiple water facilities in the displacement camp, as well as a polluted water source in the host community, to gather water. Their decision is based on total collection time, reflecting a documented preference among camp inhabitants for avoiding long queues [19,35,36]. PoCs collect water for a number of time steps, based on total collection time. Each round trip, PoCs refill their 20 L storage containers; (4) Shelters decrease water quantity in household-level containers by 0.750 L/person, and PoCs decrease their water level by 0.1875 L. These values were based on a daily use of 12 L/person for cooking and hygiene practices, as well as the consumption of 3 L/person, respectively [28]. They were calculated by dividing water quantity guidelines by the number of hours PoCs are active each day, i.e., 16; (5) Shelters contaminate water in storage containers if FRC is less than 0.1 mg/L and any PoCs belonging to them are infected, symptomatic (i.e., mild, moderate, or severe cholera infections), and do not follow adequate hygiene practices. Asymptomatic infections likely shed much less *V. cholerae*, and for a shorter amount of time [37]. We therefore assumed that these do not contribute to post-collection water contamination; (6) PoCs drink water, potentially getting exposed to *V. cholerae*; and finally (7) PoCs visit their friends and relatives in other shelters for a number of time steps based on data published by van Zandvoort et al. [38]. Severe cholera infections are admitted to the healthcare facility, depending on the number of available beds. They die with probabilities of 1% and 50% depending on whether they receive treatment or not, respectively [37], as determined by the number of available beds in the facility. A subset of model parameters is presented in Table 1. All processes and parameters are presented in detail in Appendix A.

**Table 1.** Selected model parameters in an agent-based model of cholera transmission developed on NetLogo Table 1. long description.

Parameter	Description	Value	Source
Displacement Camp Characteristics			
number-of-shelters	Number of shelters	2,048, representing around 10,000 camp inhabitants	Authors’ estimation to reach a camp population similar to that reported by Djeddah et al. [39]
health-capacity	Inpatient capacity in the health facility	20	Authors’ estimation for a population of 10,000, assuming an attack rate for clinically apparent cholera of 5% and an average length of stay of 4 days [34]
frc-initial	Free residual chlorine at water facility entities in the displacement camp	Between 0 and 1 mg/L	Set on the NetLogo Interface
water-capacity	Capacity of water facility entities in the displacement camp. This describes the state of access to safe water in the camp and affects waiting time at tap stands	Between 0.2 and 1	Set on the NetLogo Interface
hygiene-level	Percentage of camp inhabitants who follow adequate hygiene practices when provided with sufficient water	Between 0 and 100%	Set on the NetLogo Interface
scenario	Scenarios simulated through the agent-based model. These are detailed in the Experiments subsection	Displacement Camp, Acute Population Influx, Heavy Rainfall	Set on the NetLogo Interface
decrease-pp-hygiene	Percentage points decrease in *hygiene-level* in blocks housing new arrivals in the Acute Population Influx *scenario*	20	Authors’ assumption. *decrease-pp-hygiene* is included in a sensitivity analysis
capacity-blocks-arrivals	Capacity of water facility entities in blocks housing new arrivals in the Acute Population Influx *scenario*	0.2 (i.e., one functioning tap per 1,250 people)	Authors’ assumption. *capacity-blocks-arrivals* is included in a sensitivity analysis
Epidemiological Model			
exposure-probability	Probability of collecting water contaminated with *Vibrio cholerae* from an unprotected and polluted water source	0.35	Calibration on data reported by Djeddah et al. [39]
probability-symptomatic	Probability that an infected person will develop symptomatic cholera (i.e., mild, moderate, or severe)	0.25	Gangarosa and Mosley [40]
probability-gravis	Probability that an infected person will develop severe cholera	0.02	Gangarosa and Mosley [40]
Water Consumption and Collection			
walking-speed	Walking speed based on a round-trip time of 30 min over a distance of 1,000 m	0.03 min/m	Cairncross [41]
time-fill-water-container	Time required to fill water container based on a flow rate of 7.5 L/min	3 min	Authors’ estimation based on Sphere Association [28]
alternative-source-threshold	Ratio of the minimum collection time inside the displacement camp to that outside the camp, which must be exceeded to prompt camp inhabitants to collect water from the host community	2	Authors’ assumption. *alternative-source-threshold* is included in a sensitivity analysis
Social Network			
rewire-communities	Probability that a link between two shelters is severed and a new link is formed between shelters belonging to different communities within the same block	0.25	Calibration on data reported by Djeddah et al. [39]
rewire-blocks	Probability that a link between two shelters is severed and a new link is formed between shelters belonging to different blocks	0.04	Calibration on data reported by Djeddah et al. [39]

Infections in the ABM are attributed to the domain in which the exposure event occurred. We define public domain exposure as ingestion events linked to contaminated communal water sources or environments outside the household (e.g., water points and alternative public sources). We define domestic domain exposure as ingestion events linked to water contaminated due to household-level processes after collection, including storage and handling. Each simulated infection is assigned a single domain based on the exposure event that triggered the infection.

**Figure 1.**
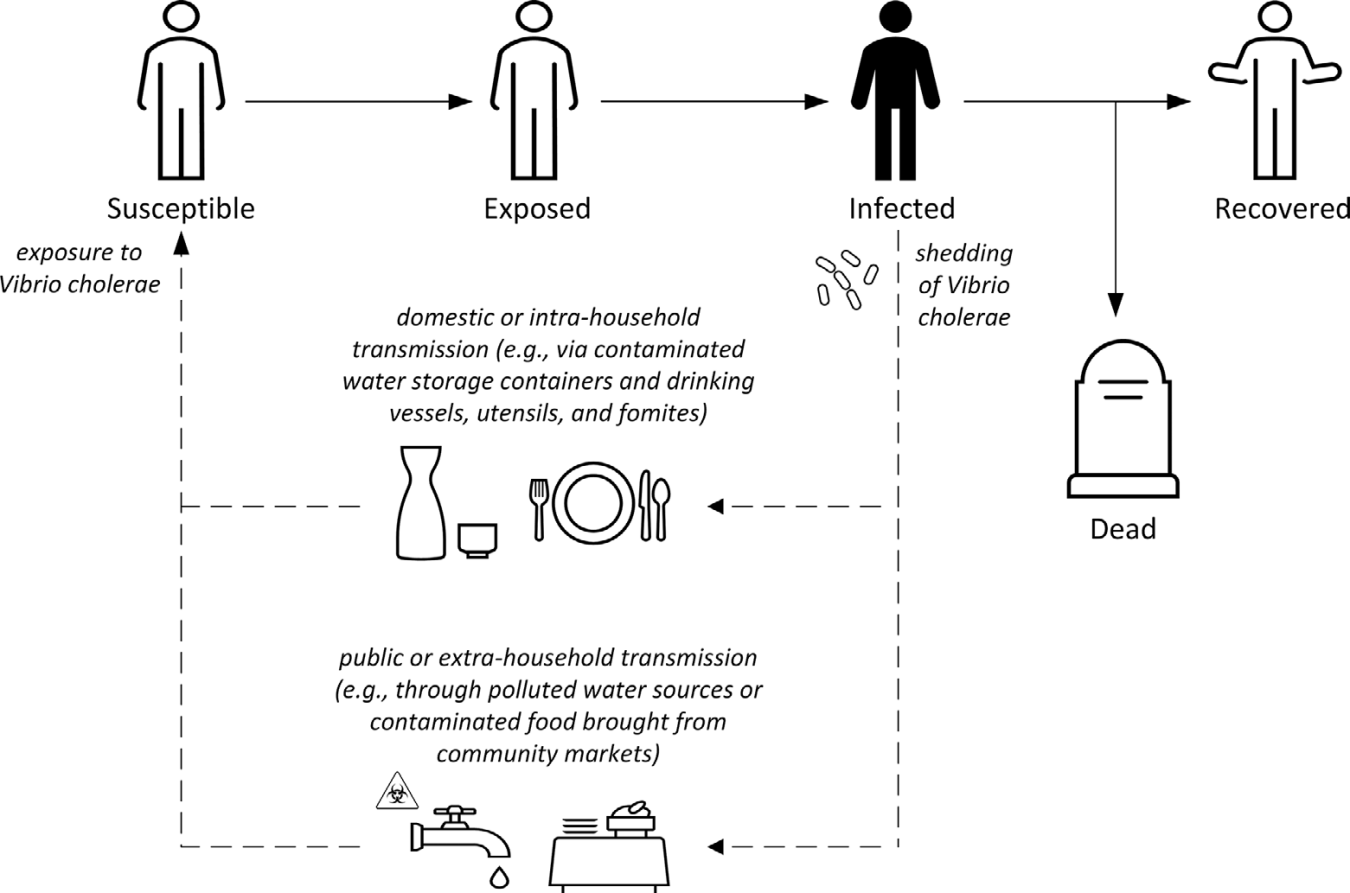
Conceptual framework of cholera transmission in the context of a displacement camp. Figure 1. long description.

In our ABM, public domain transmission is conceptualized as occurring following exposure to *V. cholerae* through an environmental point source (an unprotected and polluted water source) (Figure 1). It is governed by three parameters: *water-capacity*, *alternative-source-threshold*, and *exposure-probability* (Table 1). These determine waiting time at tap stands, the extent to which camp inhabitants prefer water delivered inside the displacement camp, and the probability of collecting contaminated water from an unprotected water source, respectively [6,21]. In contrast, domestic domain transmission is conceived as taking place following the consumption of water contaminated at the household level. It is affected by the parameters *hygiene-level* and *frc-initial*. Similar to Crooks and Hailegiorgis [26], we model exposure to *V. cholerae* as an emergent phenomenon arising from interactions among camp inhabitants and with their surrounding environment. The ABM incorporates heterogeneity in exposure to *V. cholerae* due to the location of shelters in the displacement camp. The farther the shelters are from the unprotected water source, the longer PoCs are willing to wait at tap stands, resulting in a lower probability of exposure to *V. cholerae*. It is also dependent on the hygiene practices of household members, as well as friends and relatives who may contaminate water stored in household-level containers. Moreover, the probability of exposure to *V. cholerae* through household visits is affected by varying social contact patterns. We assumed that PoCs primarily interact within their own community. This is followed by interactions in their own block, and finally, other blocks in the camp. The social network, while stylized, adds realism to the ABM and helps determine where domestic domain transmission occurs.

Through the ABM, we monitor the number of susceptible, exposed, infected, and recovered PoCs over time, as well as the cumulative number of infections and the percentage attributable to each of the two transmission domains.

### Model calibration and validation

Calibration was performed to run the ABM with plausible parameter values that represent a displacement camp, increasing confidence in its outputs (Appendix D). Three model parameters (see *exposure-probability*, *rewire-communities*, and *rewire-blocks* in Table 1) were calibrated using data from an outbreak that occurred in 1985 in a refugee camp with around 10,000 inhabitants [39]. In this outbreak, no water and sanitation response measures appear to have been carried out. Instead, emphasis reportedly lay on preparing isolation wards, treating contaminated materials, and providing patient care [39]. Moreover, with an attack rate of clinically apparent cholera (i.e., severe dehydration, vomiting, and diarrhea) above 5%, we can assume that the majority of camp inhabitants had been infected with *V. cholerae*.

The number of infections over time was not explicitly reported by Djeddah et al. [39]. Accordingly, we estimated these data in two steps. First, we visually approximated and extracted the number of cholera cases over time from the epidemic curve (see Figure 1 in [39]). Second, assuming that only 7% of those infected with *V. cholerae* El Tor develop moderate or severe cholera [40], we estimated the total number of infections by dividing the number of clinically apparent cholera cases by 0.07.

Similar to Radchuk et al. [42], we calibrated the ABM through the BehaviorSearch software tool [43]. We varied six parameters, employing a standard Genetic Algorithm (GA) against one criterion, the number of cholera infections over time (GA settings and search space specifications are available in Appendix D). The objective measure used was the sum of squared errors.

**Figure 2.**
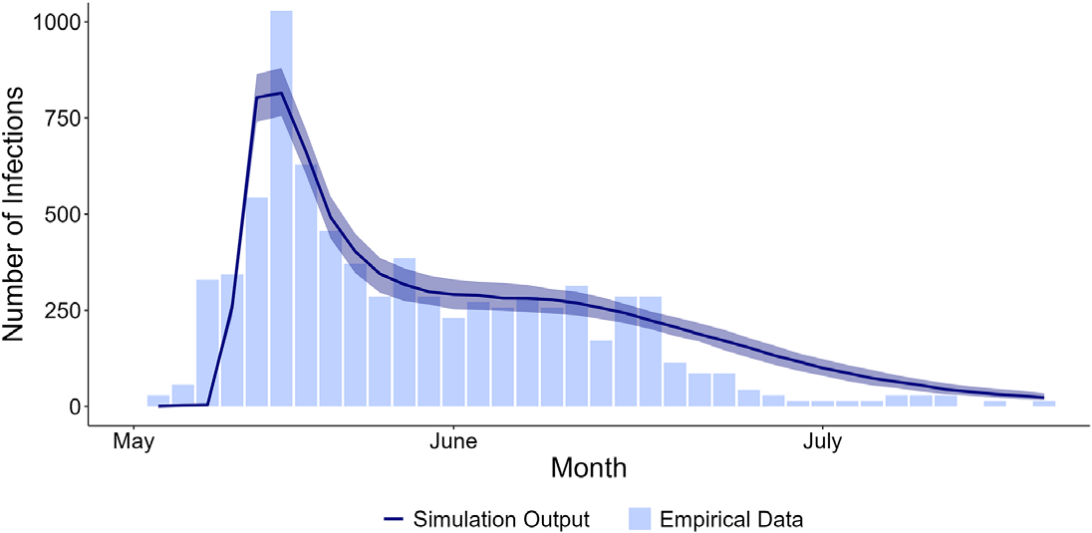
Number of cholera infections over time during an outbreak in a displacement camp. Bars are based on empirically derived data [39]. The line represents the median number of infections across model runs. Shaded areas represent the first and third quartiles. Simulation output was calculated from 400 runs with *rewire-communities* = 0.25, *rewire-blocks* = 0.04, *exposure-probability* = 0.35, *water-capacity* = 0.55, *hygiene-level* = 19, *frc-initial* = 0 mg/L, and *scenario* = ‘Displacement Camp’. Figure 2. long description.

To assess whether the ABM captures the dynamics of a cholera outbreak, we generated an epidemic curve reporting the number of infections over time from model runs with calibrated parameter values and overlayed it on the empirically derived data [39]. Through visual inspection, we found that our calibrated model reproduces phases reported in an outbreak in a displacement camp: an initial peak in cholera infections followed by a ‘plateau’ (i.e., a relatively constant number of new infections over time), and ultimately, a decrease in the number of infections until outbreak termination (Figure 2).

The ABM was informed by a rapid appraisal carried out in displacement camps in Northeast Nigeria [44]. It included semi-structured interviews, transect walks, and field observations. The rapid appraisal found that cholera outbreaks there may be facilitated by interactions between IDPs and the host community, as well as suboptimal WaSH. For instance, an outbreak that affected camps in Gwoza, Borno State in 2021 was reported to originate from a contaminated well in the host community. Moreover, 9 out of 25 (36%) water samples drawn from household-level storage containers in camps had insufficient residual chlorine, indicating that post-collection contamination may contribute to the spread of *V. cholerae*. Jaber et al. [44] also found that WaSH facilities in displacement camps were vulnerable to population influxes and flood events.

We also conducted a consultation with staff of the International Organization for Migration (IOM) regarding water collection behavior and social interactions to validate the key assumptions underlying our model.

### Experiments with the model

To explore the impact of shocks on the percentage of infections emanating from each of the two transmission domains, as well as the size and timescale of cholera outbreaks, we simulated three scenarios.

In scenario A, water supply in the displacement camp is aligned with Sphere standards, i.e., five functioning taps per 1,250 people [28]. Water drawn from the tap stands is chlorinated and can only contribute to the spread of *V. cholerae* through post-collection water contamination. As mentioned above, the ABM includes an unprotected, exogenously polluted water source located outside the camp. In scenario B, the displacement camp experiences an acute influx of arrivals. Consequently, water supply in half of the blocks is reduced to one tap per 1,250 people. Moreover, taps are allowed to malfunction (see Appendix A for more details). In this scenario, the ABM also incorporates worsened hygiene practices in blocks housing new arrivals. In scenario C, one water facility in the displacement camp is exogenously contaminated by heavy rainfall and subsequent flooding. It is set to mirror water contamination in the host community and is not affected by bacterial shedding inside the camp. These scenarios were run with varying percentages of PoCs following adequate hygiene practices and residual chlorine levels at tap stands (i.e., *hygiene-level* and *frc-initial*, respectively). *hygiene-level* varied between 0% and 80%, in increments of 40%, while *frc-initial* was set to one of two values, 0 and 0.5 mg/L, resulting in a total of 18 experiments.

In all simulation experiments, *V. cholerae* is initially introduced into the displacement camp through one infected and symptomatic PoC (i.e., the index case). It may then spread due to post-collection water contamination or the use of an unprotected, polluted water source.

### Model output analysis

We plotted the number of infections over time and the percentage of them that were due to domestic domain transmission. We also tabulated the median, 2.5th and 97.5th percentiles of the percentage of infections originating in the domestic domain, as well as four epidemic characteristics presented by Cadoni and Gaeta [45], for each of our simulation experiments. These are the epidemic peak (i.e., the maximum number of infected), the total number of infections, the time of occurrence of the peak, and the time span of the epidemic. Lastly, we performed a one-at-a-time sensitivity analysis to examine the robustness of model outcomes to variations in calibrated parameters and those selected based on the authors’ assumptions (see Table 1). These consist of six parameters. Four relate to the three scenarios. They are the ratio that must be exceeded for PoCs to seek water outside the camp, the probability that water drawn from an unprotected, polluted source is contaminated with *V. cholerae*, and the probabilities that determine link formation between communities and blocks. Two other parameters capture the extent of the influx in scenario B: water supply capacity in blocks housing new arrivals and their hygiene practices. For this sensitivity analysis, outbreaks were simulated with adequate chlorination using two values of each of these parameters. All analyses were conducted in RStudio [46] using R version 4.3.3.

The number of runs was determined in accordance with Lorscheid et al. [47]. The coefficient of variation, defined as the ratio of the sample standard deviation to its mean, was calculated for our outcomes of interest across runs of different sizes (see Appendix B for more details). These calculations were done for three parameter sets, one for each of the scenarios A, B, and C, under the most extreme simulated conditions (i.e., *hygiene-level* = 0% and *frc-initial* = 0 mg/L). We then identified the stability point for each of our outcomes [48], i.e., the sample size at which the difference between consecutive coefficients of variation fell below a criterion, set to 0.05. The maximum of these stability points was 400, representing an estimate for the minimum number of runs required for each parameter set.

## Results

### Model output

#### Scenario A: displacement camp

In scenario A, 5 out of 2,400 simulation runs did not lead to the spread of *V. cholerae* following its introduction into the camp and were excluded from all subsequent analyses (see Table 2 for detailed results by *hygiene-level* and *frc-initial*). Most outbreaks (1,793 out of 2,395 [75%]) were small, with less than 1% of camp inhabitants infected with *V. cholerae.* Also, outbreaks in 1,833 runs (77%) self-terminated before 90 days.

**Table 2.** Summary of scenario A outbreaks simulated in an agent-based model of cholera transmission developed on NetLogo Table 2. long description.

*hygiene-level; frc-initial* ^a^	Simulation runs N (%)	Small outbreaks N (%)^c^	Self-terminating outbreaks N (%)^c^
0%; 0 mg/L	400 (100)	46 (12)	46 (12)
40%; 0 mg/L	400 (100)	165 (41)	199 (50)
80%; 0 mg/L	400 (100)	390 (98)	398 (100)
0%; 0.5 mg/L	400 (100)	397 (99)	395 (99)
40%; 0.5 mg/L	397 (99)	397 (100)	397 (100)
80%; 0.5 mg/L	398 (100)	398 (100)	398 (100)
Across all *hygiene-level* and *frc-initial* values	2,395 (100)	1,793 (75)	1,833 (77)

a *hygiene-level* and *frc-initial* are parameters of the agent-based model.

b Outbreaks affecting less than 1% of camp inhabitants.

c Percentages were calculated out of included simulation runs.

**Figure 3.**
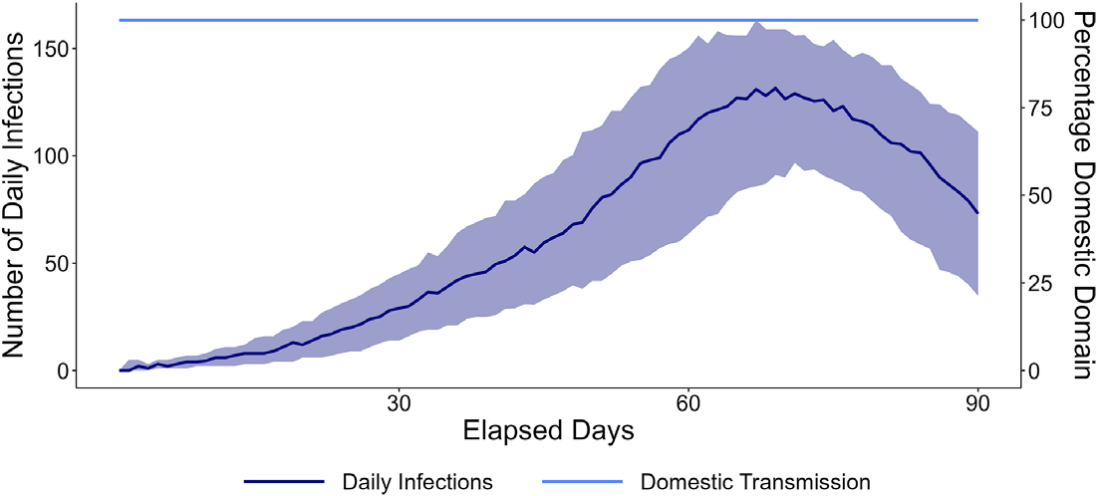
Number of daily infections over time in an agent-based model of cholera transmission in a displacement camp assuming sufficient water supply, extremely poor hygiene conditions, and inadequate chlorination (scenario A). Dark and light blue lines represent the median number of daily infections and the median percentage of infections due to domestic domain transmission, respectively. Shaded areas represent the first and third quartiles. There is no variability in percentage domestic domain across simulation runs under these assumptions. Simulation output was calculated from 400 model runs with *scenario* = ‘Displacement Camp’, *hygiene-level* = 0%, and *frc-initial* = 0 mg/L. Figure 3. long description.

Severe outbreaks did occur under extremely poor hygiene conditions and inadequate chlorination (Figure 3). These outbreaks progressed slowly, recording a peak median number of daily infections, 132 (Interquartile Range [IQR] = 68), on the 69th day. In scenario A, PoCs did not rely on the unprotected water source located outside the camp. Accordingly, there was no variability in the percentage of the domestic domain across simulation runs. The outbreaks were driven exclusively by domestic transmission.

#### Scenario B: acute population influx

**Figure 4.**
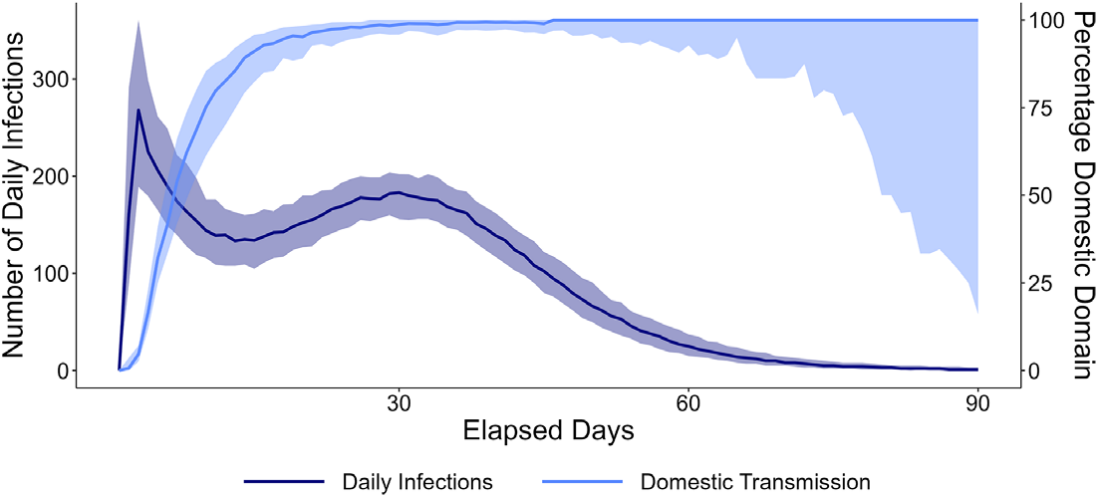
Number of daily infections over time in an agent-based model of cholera transmission in a displacement camp assuming an acute influx of arrivals, extremely poor hygiene conditions, and inadequate chlorination (scenario B). Dark and light blue lines represent the median number of daily infections and the median percentage of infections due to domestic domain transmission, respectively. Shaded areas represent the first and third quartiles. Simulation output was calculated from 400 runs with *scenario* = ‘Acute Population Influx’, *hygiene-level* = 0%, and *frc-initial* = 0 mg/L. Figure 4. long description.

None of the outbreaks in scenario B were small or self-terminated before 90 days. When hygiene conditions were abysmal and chlorination was inadequate (*hygiene-level* = 0% and *frc-initial* = 0 mg/L), cholera outbreaks were characterized by three phases: an initial peak in daily infections, followed by sustained cholera spread, and ultimately a decrease in infections. These outbreaks were initially driven by public transmission, a consequence of many PoCs relying on the unprotected water source located outside the displacement camp. Domestic transmission, however, dominated after the initial peak in infections (Figure 4). Under these circumstances, the median number of daily infections (incidence) peaked at 268 (IQR = 172) on the 3rd day.

**Figure 5.**
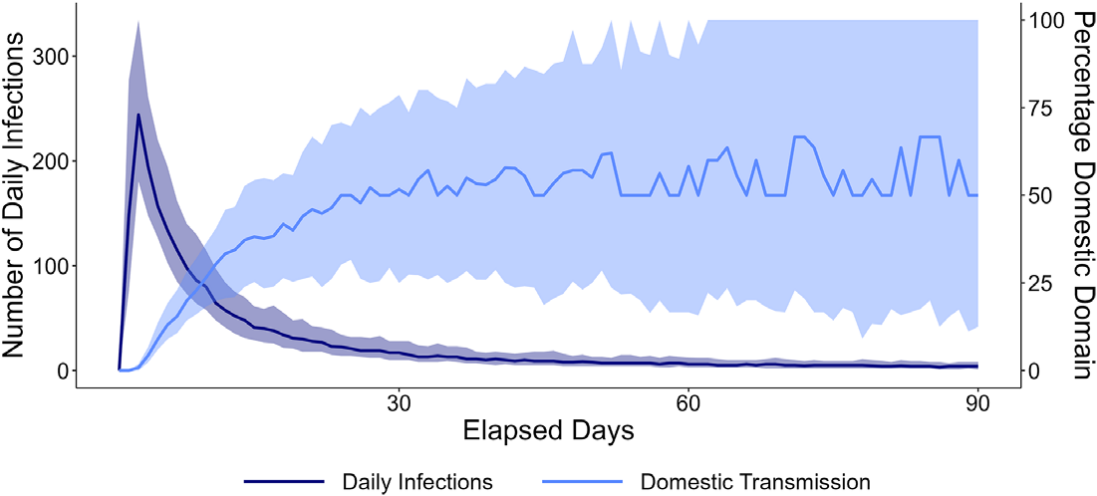
Number of daily infections over time in an agent-based model of cholera transmission in a displacement camp assuming an acute influx of arrivals, poor hygiene conditions, and adequate chlorination (scenario B). Dark and light blue lines represent the median number of daily infections and the median percentage of infections due to domestic domain transmission, respectively. Shaded areas represent the first and third quartiles. Simulation output was calculated from 400 runs with *scenario* = ‘Acute Population Influx’, *hygiene-level* = 40%, and *frc-initial* = 0.5 mg/L. Figure 5. long description.

Improved hygiene conditions (*hygiene-level* = 40%) and increased FRC at tap stands (*frc-initial* = 0.5 mg/L) markedly altered the epidemic curve: following an initial peak, the number of cholera infections sharply decreased, and the outbreaks persisted with a low number of daily infections (Figure 5). These outbreaks continued to be shaped, in part, by public transmission. The high variability in percentage domestic transmission in Figure 5 may be due to reduced daily infections and the random malfunctioning of taps.

#### Scenario C: heavy rainfall

**Figure 6.**
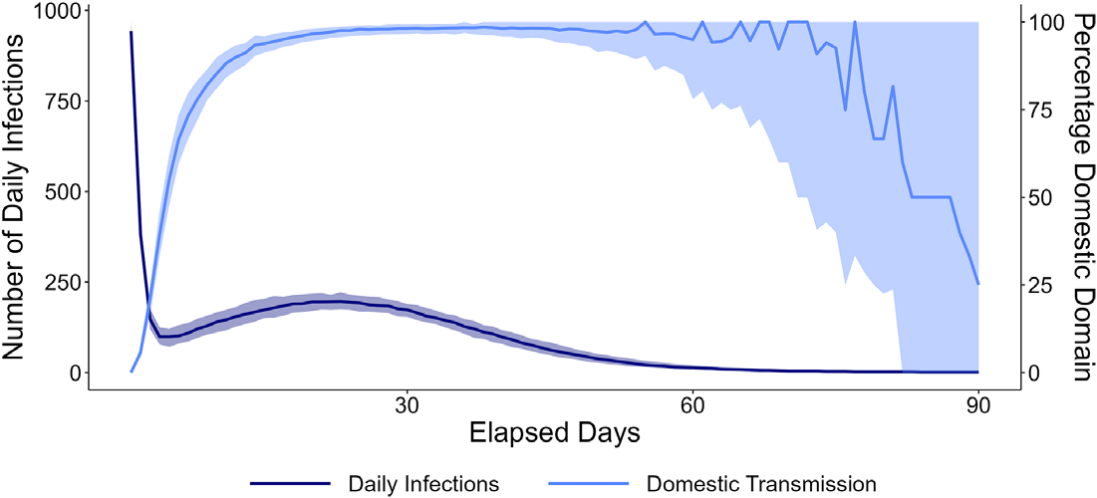
Number of daily infections over time in an agent-based model of cholera transmission in a displacement camp assuming one water facility inside the camp is contaminated following heavy rainfall, extremely poor hygiene conditions, and inadequate chlorination (scenario C). Dark and light blue lines represent the median number of daily infections and the median percentage of infections due to domestic domain transmission, respectively. Shaded areas represent the first and third quartiles. Simulation output was calculated from 400 runs with *scenario* = ‘Heavy Rainfall’, *hygiene-level* = 0%, and *frc-initial* = 0 mg/L. Figure 6. long description.

No outbreaks were small or self-terminated in scenario C. Similar to scenario B, under extremely poor hygiene conditions and in the absence of residual chlorine at tap stands, we can distinguish three phases (Figure 6). The outbreaks were initially driven by public domain transmission, with domestic transmission predominating after the initial peak in daily infections. Towards the end of the outbreaks, and as the number of daily infections decreased, public transmission once more contributed markedly to the spread of *V. cholerae*.

**Figure 7.**
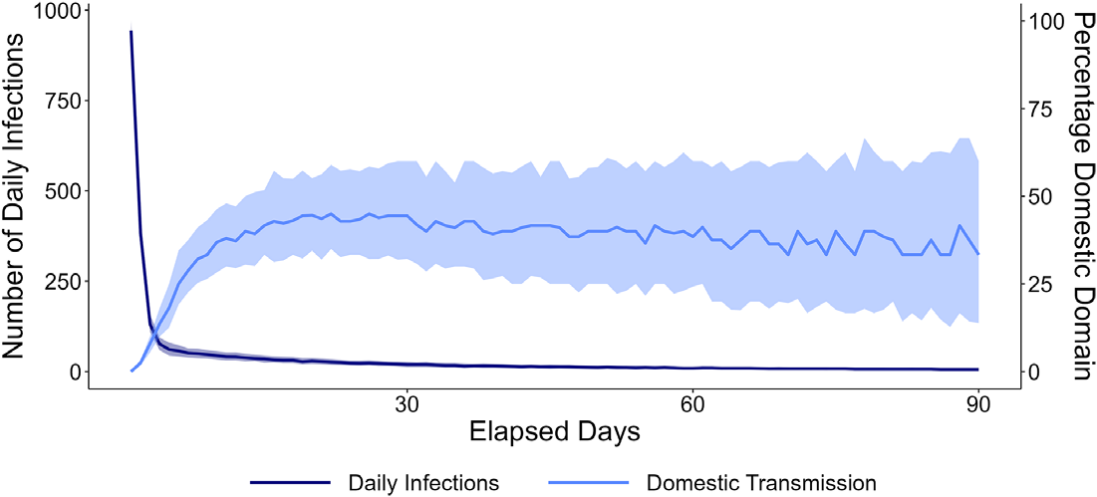
Number of daily infections over time in an agent-based model of cholera transmission in a displacement camp assuming one water facility inside the camp is contaminated following heavy rainfall, poor hygiene conditions, and adequate chlorination (scenario C). Dark and light blue lines represent the median number of daily infections and the median percentage of infections due to domestic domain transmission, respectively. Shaded areas represent the first and third quartiles. Simulation output was calculated from 400 runs with *scenario* = ‘Heavy Rainfall’, *hygiene-level* = 40%, and *frc-initial* = 0.5 mg/L. Figure 7. long description.

These outbreaks progressed faster than those under scenarios A and B, with a median peak daily infection count of 943 (IQR = 47) on the 1st day. This is due to rapid exposure of PoCs to *V. cholerae* through water delivered within the displacement camp. The adoption of adequate hygiene practices and improved chlorination substantially limited domestic transmission (Figure 7).

### Epidemic characteristics

The epidemic peak, cumulative number of infections, time of the peak, and epidemic duration for each experiment are presented in Table 3. Outbreaks in scenarios B and C were consistently large and were characterized by median epidemic peaks of 886 (IQR = 321) and 1,093 (IQR = 118), respectively. In scenario A, the median epidemic peak (8 [IQR = 24]) and median cumulative number of infections (15 [IQR = 100]) were small. However, large outbreaks occurred under poor hygiene conditions and without adequate chlorination (Table 3). The epidemic peak was reached most rapidly in scenario C, followed by scenarios B and A. Following shocks, none of the outbreaks self-terminated within 90 days, whereas in scenario A, they had a median duration of 34 days (IQR = 46).

**Table 3.** Medians, 2.5th and 97.5th percentiles of the epidemic peak, cumulative infections, time of occurrence of the peak, time span of the epidemic, and domestic domain transmission across experiments simulated in an agent-based model of cholera transmission developed on NetLogo Table 3. long description.

*scenario*	*hygiene-level; frc-initial* ^a^	Median epidemic peak N [2.5th – 97.5th Percentiles]	Median cumulative infections N [2.5th – 97.5th Percentiles]	Median elapsed time at epidemic peak Days [2.5th – 97.5th Percentiles]	Median epidemic time span Days [2.5th – 97.5th Percentiles]	Domestic domain transmission % [2.5th – 97.5th Percentiles]
A - Displacement camp	0%; 0 mg/L	868 [5–1,135]	6,410 [8–7,600]	74 [5–90]	90 [23–90]	100 [100–100]
	40%; 0 mg/L	44 [5–226]	266 [9–1,810]	32 [5–90]	90 [23–90]	100 [100–100]
	80%; 0 mg/L	11 [4–30]	22 [7–101]	9 [4–29]	32 [22–60]	100 [100–100]
	0%; 0.5 mg/L	6 [2–16]	9 [4–66]	9 [4–39]	31 [20–80]	100 [100–100]
	40%; 0.5 mg/L	5 [3–12]	8 [4–33]	8 [4–25]	27 [20–55]	100 [100–100]
	80%; 0.5 mg/L	5 [2–8]	7 [4–16]	8 [4–18]	26 [20–44]	100 [100–100]
Across *hygiene-level* and *frc-initial* values		8 [3–1,026]	15 [4–7,331]	10 [4–89]	34 [20–90]	100 [100–100]
B - Acute population influx	0%; 0 mg/L	1,134 [899–1,489]	8,376 [8,050–8,663]	29 [9–46]	90 [90–90]	81 [73–87]
	40%; 0 mg/L	948 [650–1,349]	7,222 [6,123–7,962]	10 [8–47]	90 [90–90]	75 [67–82]
	80%; 0 mg/L	832 [513–1,264]	3,919 [2,606–5,175]	10 [8–19]	90 [90–90]	45 [32–57]
	0%; 0.5 mg/L	836 [509–1,211]	3,192 [2,080–4,147]	9 [8–20]	90 [90–90]	29 [22–35]
	40%; 0.5 mg/L	803 [479–1,217]	2,998 [1,760–4,013]	10 [8–17]	90 [90–90]	22 [17–27]
	80%; 0.5 mg/L	783 [480–1,196]	2,806 [1,814–3,756]	9 [8–18]	90 [90–90]	12 [9–15]
Across *hygiene-level* and *frc-initial* values		886 [510–1,342]	3,638 [2,055–8,532]	10 [8–41]	90 [90–90]	34 [10–84]
C - Heavy rainfall	0%; 0 mg/L	1,174 [1,021–1,390]	8,312 [8,023–8,565]	7 [6–36]	90 [90–90]	77 [72–81]
	40%; 0 mg/L	1,107 [959–1,286]	6,204 [5,063–7,059]	6 [6–7]	90 [90–90]	65 [59–71]
	80%; 0 mg/L	1,074 [946–1,240]	3,198 [2,434–4,268]	6 [6–7]	90 [90–90]	23 [15–30]
	0%; 0.5 mg/L	1,084 [943–1,267]	3,546 [2,621–4,589]	6 [6–7]	90 [90–90]	33 [27–38]
	40%; 0.5 mg/L	1,082 [950–1,230]	3,088 [2,395–3,997]	6 [6–7]	90 [90–90]	20 [16–24]
	80%; 0.5 mg/L	1,067 [938–1,227]	2,767 [2,122–3,671]	6 [6–7]	90 [90–90]	7 [5–10]
Across *hygiene-level* and *frc-initial* values		1,093 [949–1,303]	3,556 [2,331–8,466]	6 [6–30]	90 [90–90]	29 [6–79]

a *scenario*, *hygiene-level*, and *frc-initial* are parameters of the agent-based model.

Outbreaks in scenario A were exclusively driven by domestic transmission. It also predominantly shaped outbreaks in scenarios B and C under poor hygiene conditions and inadequate chlorination. For example, in scenario B, the percentages of infections originating in the domestic domain were 81% (IQR = 5) and 75% (IQR = 5) when *hygiene-level* was set to 0% and 40%, respectively, absent residual chlorine at tap stands (Table 3). Chlorine markedly decreased these percentages.

### Sensitivity analysis

In scenario A, model outcomes were not sensitive to variations in the four parameters. However, in scenario B, they were highly sensitive to two parameters: *alternative-source-threshold* and *capacity-blocks-arrivals*. Their impact on outbreak dynamics is evident. First, as the preference for water delivered inside the camp increases, fewer PoCs use the unprotected water source. Under these circumstances, simulated outbreaks were smaller, and the contribution of domestic domain transmission to the spread of *V. cholerae* increased. Nonetheless, they remained predominantly shaped by public transmission. Second, increasing water supply capacity in blocks housing new arrivals mitigates water shortages and prevents the use of unprotected water sources. The outbreaks then resembled those in scenario A. Therefore, model outcomes specific to scenario B are indicative of a severely overwhelmed water supply. In scenario C, although domestic domain transmission increased slightly with more interactions between blocks, simulated outbreaks remained mainly driven by public transmission. Detailed results are available in Appendix C.

## Discussion

This study illustrates how ABMs can be used to describe cholera transmission dynamics in a stylized displacement camp and explore extreme scenarios *in silico*, supporting the design of cholera response strategies. Our ABM builds on the work of Crooks and Hailegiorgis [26], which, although very insightful, did not explicitly distinguish between and report on the two transmission domains. Moreover, it did not incorporate chlorine decay and post-collection water contamination, which may play an important role in waterborne disease transmission. Our model incorporated post-distribution chlorine decay, and explicitly investigated the contributions of each of the two transmission domains under varying conditions.

When water supply is sufficient and adequately chlorinated, cholera outbreaks simulated in our ABM are small and self-terminating. This finding is not surprising given that Shannon et al. [8] identified a notable decrease in the frequency and severity of cholera outbreaks in refugee camps, attributing it to improved cholera preparedness, multisectoral coordination, and adherence to humanitarian standards. Although WaSH minimum standards and indicators are not always predicated on published evidence [15,49], our work suggests that those related to water supply capacity (i.e., 250 people per tap) and chlorination can be effective in reducing the spread of *V. cholerae* in camps. Nevertheless, they may be insufficient when water supply is rationed, and consequently, waiting times at tap stands are much higher and water is stored for longer periods of time.

Following floods or population influxes, outbreaks are characterized by a high epidemic peak and progress rapidly. They are initially shaped by public domain transmission. Heavy rainfall and floods destroy latrines, flush fecal matter into surface water, and contaminate ground water sources, potentially exposing many camp inhabitants to *V. cholerae* [12,20,35]. Climate change exacerbates cholera outbreaks in African displacement camps by increasing the frequency of extreme weather events such as floods and droughts [9]. An unplanned population influx may overwhelm camp infrastructure [44], leading to long queues at tap stands. The subsequent reduction in water access compromises hygiene practices and leads to a reliance on unprotected water sources [36,50]. The speed at which *V. cholerae* may spread in camps when water supply is insufficient or compromised, as illustrated through the ABM, is alarming and poses considerable challenges for cholera response. This emphasizes the importance of preventing public domain transmission through environmental measures, such as source protection supported by extensive water quality monitoring prior to and during the rainy season, and health interventions such as vaccination. International WaSH guidelines provide recommendations for preventing and mitigating the consequences of rainfall and flooding, including incorporating flood risk into site selection, relying on alternative sanitation systems in flooded areas [28], and establishing burial sites away from flood-prone or waterlogged areas [51]. While vaccines have been deployed in camps in response to cholera outbreaks [52,53], two-dose campaigns require several weeks to achieve full protection of all camp inhabitants [54] and may therefore be of limited effectiveness in the wake of a shock, unless conducted pre-emptively.

In our ABM, outbreaks in displacement camps that meet the minimum water supply standards are exclusively driven by domestic transmission. More interesting, however, is that domestic transmission may underlie outbreak dynamics in those that do not. Hence, its role in sustaining or exacerbating cholera outbreaks cannot be overstated. Domestic domain transmission, mediated among other means by contaminated storage containers [55], has contributed to the spread of *V. cholerae* in past outbreaks. For instance, Phelps et al. [56] demonstrated the role of household-level transmission in the spread of cholera during an outbreak in Copenhagen in 1853. Moreover, during an outbreak in Peru in 1991, Swerdlow et al. [57] found that water quality deteriorated during distribution and storage, with the highest fecal coliform counts observed in household-level storage containers. Among forcibly displaced people, multiple risk factors in the domestic domain were identified through investigations carried out in refugee camps in Kenya [17,18,21]. Our work suggests that domestic transmission ought not be overlooked even when inhabitants may be exposed to *V. cholerae* through a common source, especially absent adequate hygiene practices or chlorination. Domestic transmission could be prevented through various targeted interventions, such as the provision of improved water storage containers, e.g., with narrow neck or faucet, or monitoring of chlorine levels [29]. Which interventions are most suitable for a certain situation in terms of acceptability and effectiveness, needs to be ascertained. Such efforts could be supported through risk communication and community engagement (RCCE) campaigns to reinforce hygiene measures, such as handwashing with soap [18], which may also reduce food-borne transmission.

It is hard to assess trade-offs and determine which domain to focus on in a certain context, also because various interventions tend to be effective at different time scales. Transmission in the public domain can most effectively be targeted through preventive measures. Our ABM underscores that outbreak response strategies need to be contextualized and adaptive. It also demonstrates that even following shocks or under conditions of environmental contamination or insufficient WaSH facilities, interventions targeting domestic domain transmission may help contain the spread of *V. cholerae* and reduce outbreak severity. However, this may not be the case under more severe water shortage or if all water delivered in a displacement camp is contaminated.

There are some limitations inherent to our study. First, the ABM does not explicitly account for foodborne transmission, even though it may contribute to the spread of cholera [58]. We assumed that post-collection water contamination acts as a proxy to local fecal contamination, allowing us to partly capture the dynamics of intra-household transmission of *V. cholerae* through other means. Inclusion of foodborne transmission would, to some extent, amplify transmission in the domestic domain [4,22]. Second, in absence of in-depth research on water collection behavior in displacement camps, water collection decision making was only based on total collection time. While it constitutes a limitation, the ABM does incorporate two preferences among camp inhabitants, one for avoiding long queues, and another for water delivered inside the displacement camp. Third, we modeled a stylized camp based on multiple UNHCR and Sphere minimum standards [28,33]. To study cholera outbreaks in informal camps, parameters in the model may have to be adjusted. The ABM would also need to be modified to account for different dynamics. For example, the camp may be less structured, which could have implications for water collection behavior and social interactions. Assumptions regarding water supply may also need to be adjusted. This would be a good topic for future research. The current findings cannot be generalized to other types of humanitarian crises and should be understood in the context of a planned displacement camp. Fourth, a single rather old data set [39] was used for calibration. While we recognize that these data entail a risk of being outdated, in absence of adequate recent data we consider them suitable enough for our ABM. They are sufficiently representative to simulate uncontrolled cholera outbreaks in a stylized camp without any water and sanitation response measures. Recent cholera outbreak data from displacement camps would allow us to update the model and refine its parameters.

Our ABM could be further developed in various other ways as well. For instance, it would be good to expand the water quantity dimension and explore more thoroughly how reduced access to water constrains household hygiene behavior. When people shift to alternative sources, they not only face potentially worse water quality. They may use less water overall because it must be carried further. Future work could also be informed by behavioral surveys on water collection and use conducted in displacement camps. Another improvement of the model could be to quantify the infective dose by distinguishing between more or less infective individuals, include concentrations of *V. cholerae* in the water sources, or account for multiple exposure pathways.

The model can be expanded to simulate intervention scenarios in the public and domestic transmission domains. We could incorporate vaccination campaigns, water chlorination at points of distribution or water trucking, hygiene behavior, the effects of RCCE campaigns, or household water purification to assess to what extent these would affect the two domains. This would support the development of context-specific recommendations.

ABMs like ours can be used to investigate the relative importance of the public and domestic domains in the transmission of other infectious diseases associated with overcrowding, poor water infrastructure or overwhelmed health services, e.g., diarrheal diseases [13,14], respiratory infections [38,59] or poliomyelitis [60]. Such modeling efforts could assess the impact of external shocks such as flooding, population influx, or devastating epidemics of other infectious diseases [61], and simulate domain-specific intervention scenarios.

## Conclusion

The spread of *V. cholerae* in camps demonstrates varying dynamics across the contributions of the two transmission domains, as well as the outbreak size and timescale. Domestic transmission may exclusively shape outbreaks in displacement camps, specifically when the water supply meets humanitarian standards. However, these outbreaks appear to be small, unless hygiene conditions are poor and water is not adequately chlorinated. In such instances, we recommend focusing on controlling intra-household fecal-oral contamination, for example, through hygiene promotion activities, the provision of narrow neck containers, and the prioritization of free residual chlorine monitoring at tap stands and in household water containers. To prevent large outbreaks of *V. cholerae*, humanitarian activities ought to be directed towards mitigating the consequences of extreme weather events and unplanned population influxes on WaSH infrastructure. Following shocks, outbreaks progress rapidly and are characterized by a high epidemic peak. While they are initially shaped by public domain transmission, the domestic domain may drive the spread of *V. cholerae* after an initial surge in infections. Cholera response strategies need to adapt to context-specific transmission dynamics, as well as explicitly and comprehensively address extreme weather events and unplanned population influxes. Future research can support the development of such strategies through the use of ABMs to explore the effectiveness of alternative intervention packages and delivery strategies, and the synergies between WaSH measures and vaccination campaigns.

## Supporting information

10.1017/S0950268826101575.sm001 Jaber et al. supplementary material Jaber et al. supplementary material

## Data Availability

The authors confirm that the data supporting the findings of this study are available within the article and its supplementary materials.

## Supplementary material

The supplementary material for this article can be found at http://doi.org/10.1017/S0950268826101575.

## Long descriptions

### Table 1 Long description

From top to bottom, the table is divided into four sections. The first section, Displacement Camp Characteristics, lists: number-of-shelters (2,048, representing about 10,000 inhabitants), health-capacity (20), frc-initial (between 0 and 1 mg per L), water-capacity (between 0.2 and 1), hygiene-level (between 0 and 100 percent), scenario (Displacement Camp, Acute Population Influx, Heavy Rainfall), decrease-pp-hygiene (20), and capacity-blocks-arrivals (0.2, one functioning tap per 1,250 people). The second section, Epidemiological Model, includes: exposure-probability (0.35), probability-symptomatic (0.25), and probability-gravis (0.02). The third section, Water Consumption and Collection, lists: walking-speed (0.03 min per m), time-fill-water-container (3 min), and alternative-source-threshold (2). The fourth section, Social Network, includes: rewire-communities (0.25) and rewire-blocks (0.04). Each parameter is accompanied by a description, its value or range, and the source or method of estimation.

Navigate back to Table 1.

### Figure 1 Long description

From left to right, the flowchart begins with a Susceptible person, labeled ‘exposure to Vibrio cholerae.’ An arrow leads to Exposed, then to Infected, depicted with bacteria and labeled ‘shedding of Vibrio cholerae.’ Two dashed arrows extend downward from Infected: the upper arrow describes domestic or intra-household transmission, with icons for water containers, utensils, and plates; the lower arrow describes public or extra-household transmission, with icons for a faucet, warning sign, and market food. From Infected, a solid arrow leads to Dead, represented by a gravestone, and another arrow leads to Recovered, depicted by a person with raised arms. All text labels are transcribed exactly as shown.

Navigate back to Figure 1.

### Figure 2 Long description

The x axis is labeled Month, spanning May, June, and July. The y axis is labeled Number of Infections, ranging from 0 to 1,000. Light blue vertical bars are based on empirically derived data, showing a sharp rise in infections in late May, peaking near 1,000, then gradually decreasing through June and July. A dark blue line represents simulation output, closely following the empirical trend, with a steep increase, peak, and subsequent decline. The shaded area around the line indicates the first and third quartiles from 400 simulation runs.

Navigate back to Figure 2.

### Table 2 Long description

The table contains seven rows and four columns. The first column lists hygiene-level and frc-initial pairs: 0 percent and 0 mg per L, 40 percent and 0 mg per L, 80 percent and 0 mg per L, 0 percent and 0.5 mg per L, 40 percent and 0.5 mg per L, 80 percent and 0.5 mg per L, and a summary row for all values. The second column shows simulation runs, always 400 or 397 or 398 per scenario, with 2,395 total. The third column shows small outbreaks, defined as affecting less than 1 percent of camp inhabitants, with counts and percentages: 46 (12), 165 (41), 390 (98), 397 (99), 397 (100), 398 (100), and 1,793 (75) overall. The fourth column shows self-terminating outbreaks, with values: 46 (12), 199 (50), 398 (100), 395 (99), 397 (100), 398 (100), and 1,833 (77) overall. Increasing hygiene-level and initial free residual chlorine from 0 to 80 percent and 0 to 0.5 mg per L results in a higher proportion of small and self-terminating outbreaks. All percentages are calculated out of included simulation runs.

Navigate back to Table 2.

### Figure 3 Long description

The x axis is labeled Elapsed Days, ranging from 1 to 90. The left y axis shows Number of Daily Infections from 0 to 150, and the right y axis shows Percentage Domestic Domain from 0 to 100. The scenario models sufficient water supply, 0 percent hygiene level, and 0 milligrams per liter initial chlorination. The dark blue line represents the median daily infections, which rise steadily from near zero, peaking between days 60 and 70 above 100, then declining toward day 90. The shaded region around this line indicates the first and third quartiles based on 400 simulation runs. The light blue line, plotted at the top of the graph, represents the median percentage of infections due to domestic domain transmission, remaining constant at 100 percent throughout the period. There is no variability in domestic transmission percentage across simulation runs.

Navigate back to Figure 3.

### Figure 4. Long description

The X-axis represents Elapsed Days with markers at 30, 60, and 90. The left Y-axis measures Number of Daily Infections from 0 to 300. The right Y-axis measures Percentage Domestic Domain from 0 to 100.

* The Daily Infections line (dark blue) rises sharply to a peak of approximately 270 within the first 10 days. It then drops to around 130 before rising to a second, broader peak of nearly 200 at day 30. After day 30, it follows a steady asymptotic curve downward, reaching near zero by day 90. A shaded dark blue region indicates the confidence interval around this line.

* The Domestic Transmission line (light blue) starts at zero and rises steeply, reaching nearly 100 percent by day 20. It remains at a plateau near 100 percent until approximately day 70, after which the shaded confidence interval expands significantly downward, while the primary line remains high.

A legend at the bottom identifies the dark blue line as Daily Infections and the light blue line as Domestic Transmission.

Navigate back to Figure 4..

### Figure 5 Long description

The x axis is labeled Elapsed Days, ranging from 1 to 90. The left y axis is Number of Daily Infections, spanning 0 to 350. The right y axis is Percentage Domestic Domain, spanning 0 to 100. Two lines are plotted with median values: a dark blue line for daily infections and a light blue line for domestic transmission percentage. The scenario models acute population influx, 40 percent hygiene level, and 0.5 milligrams per liter initial chlorination. The dark blue line peaks sharply near day 2 above 200 infections, then declines rapidly and stabilizes below 50 infections after day 30. The light blue line rises quickly, reaching about 50 percent by day 30, then fluctuates between 50 and 75 percent for the remainder of the period. Shaded areas around each line represent the first and third quartiles from 400 simulation runs.

Navigate back to Figure 5.

### Figure 6 Long description

The x axis is labeled Elapsed Days, ranging from 1 to 90. The left y axis is Number of Daily Infections, ranging from 0 to 1,000. The right y axis is Percentage Domestic Domain, ranging from 0 to 100. Two lines are plotted: a dark blue line for Daily Infections and a light blue line for Domestic Transmission. Both lines show median values, with shaded areas representing the first and third quartiles from 400 simulation runs. The scenario models heavy rainfall, 0 percent hygiene level, and 0 milligrams per liter initial chlorination. At day 1, daily infections spike near 1,000, then drop sharply and stabilize around 200, followed by a gradual decline to near zero by day 90. The percentage of domestic transmission rises rapidly to nearly 100 percent by day 15, remains high until about day 70, then declines steeply to 25 percent by day 90.

Navigate back to Figure 6.

### Figure 7 Long description

The x-axis is labeled Elapsed Days, ranging from 1 to 90. The left y-axis shows Number of Daily Infections from 0 to 1,000, and the right y-axis shows Percentage Domestic Domain from 0 to 100. Two lines are plotted: a dark blue line for Daily Infections and a light blue line for Domestic Transmission. Both lines start at day 1. The scenario models heavy rainfall, 40 percent hygiene level, and 0.5 milligrams per liter initial chlorination. The Daily Infections line spikes near 1,000 at day 1, drops sharply by day 5, then gradually declines and stabilizes below 30 infections. The Domestic Transmission line starts near zero, rises to around 40 percent by day 15, and remains relatively stable for the remainder of the period. Shaded regions around each line represent the first and third quartiles from 400 simulation runs.

Navigate back to Figure 7.

### Table 3 Long description

The table is divided into three main scenarios listed vertically: A - Displacement camp, B - Acute population influx, and C - Heavy rainfall. For each scenario, rows represent combinations of hygiene-level (0 percent, 40 percent, 80 percent) and initial free residual chlorine (frc-initial, 0 mg per L or 0.5 mg per L). Columns, from left to right, are: scenario, hygiene-level and frc-initial, median epidemic peak with 2.5th to 97.5th percentiles, median cumulative infections with percentiles, median elapsed time at epidemic peak in days with percentiles, median epidemic time span in days with percentiles, and domestic domain transmission in percent with percentiles.

In scenario A, the highest epidemic peak (868 [5–1,135]) and cumulative infections (6,410 [8–7,600]) occur at 0 percent hygiene and 0 mg per L chlorine, with both values dropping sharply as hygiene and chlorine increase, reaching a median peak of 5 [2–8] and cumulative infections of 7 [4–16] at 80 percent hygiene and 0.5 mg per L chlorine. The epidemic duration shortens from 90 [23–90] days to 26 [20–44] days. Domestic domain transmission remains at 100 percent in all cases.

In scenario B, the highest values are at 0 percent hygiene and 0 mg per L chlorine (epidemic peak 1,134 [899–1,489], cumulative infections 8,376 [8,050–8,663]), decreasing with higher hygiene and chlorine to a median peak of 783 [480–1,196] and cumulative infections of 2,806 [1,814–3,756] at 80 percent hygiene and 0.5 mg per L chlorine. Domestic domain transmission drops from 81 [73–87] percent to 12 [9–15] percent as conditions improve.

In scenario C, the highest epidemic peak is 1,174 [1,021–1,390] and cumulative infections 8,312 [8,023–8,565] at 0 percent hygiene and 0 mg per L chlorine, decreasing to 1,067 [938–1,227] and 2,767 [2,122–3,671] at 80 percent hygiene and 0.5 mg per L chlorine. Domestic domain transmission falls from 77 [72–81] percent to 7 [5–10] percent. Across all scenarios, increasing hygiene-level and chlorine consistently reduces epidemic peak, cumulative infections, and domestic transmission. Summary rows at the bottom of each scenario block show median values across all hygiene and chlorine combinations.

Navigate back to Table 3.
