# Supplementary material for: Relative contributions of public and domestic transmission domains in cholera outbreaks in displacement camps: an exploratory agent-based modeling study
Source: Epidemiol Infect. 2026 May 13;154:e97. doi: 10.1017/S0950268826101575 (PMC13366369; doi:10.1017/S0950268826101575)
Supplement: Jaber et al. supplementary material [file S0950268826101575sup001.zip › Appendix_B.docx]

Relative contributions of public and domestic transmission domains in cholera outbreaks in displacement camps: an exploratory agent-based modelling study

Appendix B - Minimum number of runs

The mean and coefficient of variation for the epidemic peak, the total number of infections, the time of occurrence of the peak, the time span of the epidemic, and the percentage of infections originating in the domestic domain were calculated from samples of varying size. These are presented below in Table 1.

Table 1: Means and coefficients of variation calculated from sets of runs of varying size in an agent-based model of cholera transmission developed on NetLogo.

| N | Epidemic Peak | | Elapsed Time at Epidemic Peak | | Cumulative Infections | | Epidemic Time Span | | Domestic Domain Transmission | |  |
| --- | --- | --- | --- | --- | --- | --- | --- | --- | --- | --- | --- |
|  | *scenario* = ‘Displacement Camp’, *hygiene-level* = 0%, and *frc-initial* = 0 mg/L | | | | | | | | | |  |
|  | MEAN | COEFVAR | MEAN | COEFVAR | MEAN | COEFVAR | MEAN | COEFVAR | MEAN | COEFVAR |  |
| 2 | 918 | 0.1101 | 80 | 0.1493 | 6629 | 0.1124 | 90 | 0 | **100** | **0** |  |
| 10 | 916 | 0.1023 | 76 | 0.1602 | 6579 | 0.1258 | 90 | 0 | 100 | 0 |  |
| 100 | 826 | 0.2788 | 74 | 0.2333 | 5862 | 0.2917 | 87 | 0.1478 | 100 | 0 |  |
| 200 | 770 | 0.4267 | 69 | 0.3507 | 5396 | 0.4385 | 83 | 0.2424 | 100 | 0 |  |
| 300 | 807 | 0.3593 | 71 | 0.2905 | 5745 | 0.3609 | 85 | 0.19 | 100 | 0 |  |
| 400 | **762** | **0.4419** | **69** | **0.3647** | **5319** | **0.4582** | **82** | **0.2452** | 100 | 0 |  |
| 800 | 782 | 0.4006 | 69 | 0.3321 | 5572 | 0.4123 | 84 | 0.2232 | 100 | 0 |  |
| 1200 | 780 | 0.3978 | 70 | 0.3279 | 5540 | 0.4104 | 84 | 0.2248 | 100 | 0 |  |
|  | *scenario* = ‘Acute Population Influx’, *hygiene-level* = 0%, and *frc-initial* = 0 mg/L | | | | | | | | | |  |
| 2 | | 966 | 0.0446 | 44 | 0.1463 | **8370** | **0.0195** | **90** | **0** | **81** | **0.0567** |
| 10 | | 1211 | 0.0411 | 21 | 0.569 | 8380 | 0.0143 | 90 | 0 | 79 | 0.033 |
| 100 | | **1126** | **0.1206** | 26 | 0.4686 | 8356 | 0.0188 | 90 | 0 | 81 | 0.0373 |
| 200 | | 1149 | 0.1218 | **25** | **0.5114** | 8361 | 0.0208 | 90 | 0 | 81 | 0.041 |
| 300 | | 1147 | 0.1221 | 23 | 0.5509 | 8358 | 0.0198 | 90 | 0 | 80 | 0.0391 |
| 400 | | 1142 | 0.1233 | 25 | 0.5374 | 8362 | 0.0192 | 90 | 0 | 80 | 0.0415 |
| 800 | | 1157 | 0.1278 | 23 | 0.5415 | 8383 | 0.0198 | 90 | 0 | 80 | 0.0408 |
| 1200 | | 1147 | 0.1225 | 24 | 0.5303 | 8362 | 0.0204 | 90 | 0 | 80 | 0.0396 |
|  | | *scenario* = ‘Heavy Rainfall’, *hygiene-level* = 0%, and *frc-initial* = 0 mg/L | | | | | | | | | |
| 2 | | **1098** | **0.0638** | 6 | 0 | **8153** | **0.0335** | **90** | **0** | **77** | **0.0099** |
| 10 | | 1254 | 0.0743 | 20 | 0.5956 | 8346 | 0.0128 | 90 | 0 | 76 | 0.0297 |
| 100 | | 1198 | 0.0971 | **17** | **0.6705** | 8314 | 0.0185 | 90 | 0 | 77 | 0.0274 |
| 200 | | 1198 | 0.0913 | 17 | 0.6505 | 8330 | 0.0171 | 90 | 0 | 77 | 0.03 |
| 300 | | 1217 | 0.0878 | 17 | 0.6419 | 8345 | 0.0171 | 90 | 0 | 76 | 0.0289 |
| 400 | | 1199 | 0.0921 | 18 | 0.653 | 8316 | 0.0182 | 90 | 0 | 77 | 0.0287 |
| 800 | | 1207 | 0.0891 | 17 | 0.6504 | 8332 | 0.0181 | 90 | 0 | 76 | 0.0298 |
| 1200 | | 1202 | 0.0895 | 17 | 0.6419 | 8319 | 0.0176 | 90 | 0 | 77 | 0.0291 |

* For each of our five outcomes, values corresponding to the sample size at which the difference between consecutive coefficients of variation falls below 0.05 are highlighted in **bold**.
