# Supplementary material for: Relative contributions of public and domestic transmission domains in cholera outbreaks in displacement camps: an exploratory agent-based modeling study
Source: Epidemiol Infect. 2026 May 13;154:e97. doi: 10.1017/S0950268826101575 (PMC13366369; doi:10.1017/S0950268826101575)
Supplement: Jaber et al. supplementary material [file S0950268826101575sup001.zip › Appendix_C.docx]

Relative contributions of public and domestic transmission domains in cholera outbreaks in displacement camps: an exploratory agent-based modelling study

Appendix C - Sensitivity analysis

A one-at-a-time sensitivity analysis was conducted to explore model robustness to variations in six parameters. We ran the model using two values of each of the parameters and assessed our five outcomes of interest for all three scenarios with *hygiene-level* set to 40% and *frc-initial* to 0.5 mg/L. Medians, as well as 2.5^th^ and 97.5^th^ percentiles, are presented in Table 1, Table 2, and Table 3. These results are based on 400 runs for each parameter set.

Table 1: Medians, 2.5^th^ and 97.5^th^ percentiles of epidemic characteristics in a one-at-a-time sensitivity analysis for scenario A.

| Parameter | Value | Median Epidemic Peak  N [2.5^th^ - 97.5^th^ Percentiles] | Median Cumulative Infections  N [2.5^th^ - 97.5^th^ Percentiles] | Median Elapsed Time at Epidemic Peak  Days [2.5^th^ - 97.5^th^ Percentiles] | Median Epidemic Time Span  Days [2.5^th^ - 97.5^th^ Percentiles] | Domestic Domain Transmission (%) [2.5^th^ - 97.5^th^ Percentiles] |
| --- | --- | --- | --- | --- | --- | --- |
| hygiene-level; frc-initial^1^ | 40%; 0.5 mg/L | 5 [3 – 12] | 8 [4 – 33] | 8 [4 – 25] | 27 [20 – 55] | 100 [100 – 100] |
| alternative-source-threshold | 1.5 | 5 [2 – 12] | 7 [3 – 32] | 8 [4 – 25] | 28 [19 – 57] | 100 [100 – 100] |
|  | 2.5 | 5 [3 – 11] | 7 [4 – 31] | 8 [4 – 25] | 27 [20 – 57] | 100 [100 – 100] |
| exposure-probability | 0.20 | 5 [3 – 14] | 7 [4 – 36] | 8 [4 – 31] | 28 [20 – 60] | 100 [100 – 100] |
|  | 0.50 | 5 [3 – 12] | 8 [4 – 32] | 8 [4 – 25] | 28 [20 – 54] | 100 [100 – 100] |
| rewire-communities | 0.10 | 5 [3 – 12] | 8 [4 – 33] | 8 [4 – 25] | 28 [20 – 57] | 100 [100 – 100] |
|  | 0.40 | 5 [3 – 13] | 8 [4 – 39] | 8 [4 – 30] | 27 [20 – 59] | 100 [100 – 100] |
| rewire-blocks | 0.01 | 5 [3 – 11] | 8 [4 – 32] | 8 [4 – 23] | 27 [20 – 55] | 100 [100 – 100] |
|  | 0.07 | 5 [3 – 11] | 8 [4 – 30] | 8 [4 – 27] | 28 [20 – 57] | 100 [100 – 100] |

^1^ Outcomes are calculated from 400 runs with *scenario* ‘Displacement Camp’, *hygiene-level* 40%, and *frc-initial* to 0.5 mg/L.

Table 2: Medians, 2.5^th^ and 97.5^th^ percentiles of epidemic characteristics in a one-at-a-time sensitivity analysis for scenario B.

| Parameter | Value | Median Epidemic Peak  N [2.5^th^ - 97.5^th^ Percentiles] | Median Cumulative Infections  N [2.5^th^ - 97.5^th^ Percentiles] | Median Elapsed Time at Epidemic Peak  Days [2.5^th^ - 97.5^th^ Percentiles] | Median Epidemic Time Span  Days [2.5^th^ - 97.5^th^ Percentiles] | Domestic Domain Transmission (%) [2.5^th^ - 97.5^th^ Percentiles] |
| --- | --- | --- | --- | --- | --- | --- |
| hygiene-level; frc-initial^1^ | 40%; 0.5 mg/L | 803 [479 – 1217] | 2998 [1760 – 4013] | 10 [8 – 17] | 90 [90 – 90] | 22 [17 – 27] |
| alternative-source-threshold^2^ | 1.5 | **1624 [1028 – 2376]** | **5428 [4011 – 7612]** | **9 [8 – 12]** | **90 [90 – 90]** | **20 [17 – 23]** |
|  | 2.5 | **87 [4 – 389]** | **534 [6 – 1362]** | **18 [7 – 66]** | **90 [24 – 90]** | **33 [21 – 100]** |
| exposure-probability | 0.20 | 612 [353 – 906] | 2700 [1708 – 3640] | 11 [8 – 22] | 90 [90 – 90] | 25 [21 – 30] |
|  | 0.50 | 942 [546 – 1410] | 3256 [2047 – 4183] | 9 [7 – 16] | 90 [90 – 90] | 20 [15 – 26] |
| rewire-communities | 0.10 | 803 [493 – 1256] | 3012 [1941 – 3997] | 10 [8 – 18] | 90 [90 – 90] | 22 [17 – 26] |
|  | 0.40 | 806 [500 – 1174] | 2950 [1910 – 3962] | 10 [8 – 18] | 90 [90 – 90] | 22 [17 – 27] |
| rewire-blocks | 0.01 | 808 [451 – 1232] | 2869 [1845 – 3749] | 10 [8 – 17] | 90 [90 – 90] | 20 [14 – 25] |
|  | 0.07 | 813 [487 – 1186] | 3156 [1927 – 4114] | 9 [8 – 16] | 90 [90 – 90] | 24 [19 – 28] |
| decrease-pp-hygiene | 0 | 806 [441 – 1188] | 2943 [1885 – 3883] | 10 [8 – 18] | 90 [90 – 90] | 19 [14 – 24] |
|  | 40 | 810 [454 – 1237] | 3016 [2024 – 4040] | 10 [8 – 19] | 90 [90 – 90] | 24 [19 – 31] |
| capacity-blocks-arrivals^2^ | 0.1 | **1894 [1348 – 2450]** | **5501 [4113 – 7025]** | **8 [7 – 9]** | **90 [90 – 90]** | **19 [16 – 22]** |
|  | 0.3 | **5 [3 – 45]** | **8 [4 – 157]** | **9 [4 – 39]** | **30 [19 – 90]** | **100 [46 – 100]** |

^1^ Outcomes are calculated from 400 runs with *scenario* ‘Acute Population Influx’, *hygiene-level* 40%, and *frc-initial* to 0.5 mg/L.

^2^ The model is highly sensitive to *alternative-source-threshold* and *capacity-blocks-arrivals*. Outcomes for these parameters are highlighted in **bold**.

Table 3: Medians, 2.5^th^ and 97.5^th^ percentiles of epidemic characteristics in a one-at-a-time sensitivity analysis for scenario C.

| Parameter | Value | Median Epidemic Peak  N [2.5^th^ - 97.5^th^ Percentiles] | Median Cumulative Infections  N [2.5^th^ - 97.5^th^ Percentiles] | Median Elapsed Time at Epidemic Peak  Days [2.5^th^ - 97.5^th^ Percentiles] | Median Epidemic Time Span  Days [2.5^th^ - 97.5^th^ Percentiles] | Domestic Domain Transmission (%) [2.5^th^ - 97.5^th^ Percentiles] |
| --- | --- | --- | --- | --- | --- | --- |
| hygiene-level; frc-initial^1^ | 40%; 0.5 mg/L | 1082 [950 – 1230] | 3088 [2395 – 3997] | 6 [6 – 7] | 90 [90 – 90] | 20 [16 – 24] |
| alternative-source-threshold | 1.5 | 1083 [947 – 1243] | 3140 [2330 – 4135] | 6 [6 – 7] | 90 [90 – 90] | 20 [16 – 24] |
|  | 2.5 | 1070 [949 – 1249] | 3069 [2295 – 4059] | 6 [6 – 7] | 90 [90 – 90] | 20 [16 – 24] |
| exposure-probability | 0.20 | 982 [885 – 1111] | 2793 [2133 – 3609] | 7 [6 – 7] | 90 [90 – 90] | 21 [17 – 25] |
|  | 0.50 | 1146 [983 – 1352] | 3388 [2538 – 4351] | 6 [6 – 7] | 90 [90 – 90] | 20 [16 – 24] |
| rewire-communities | 0.10 | 1068 [955 – 1248] | 2996 [2370 – 3952] | 6 [6 – 7] | 90 [90 – 90] | 20 [16 – 23] |
|  | 0.40 | 1082 [953 – 1255] | 3169 [2297 – 4187] | 6 [6 – 7] | 90 [90 – 90] | 20 [16 – 24] |
| rewire-blocks | 0.01 | 1062 [930 – 1221] | 2780 [2106 – 3741] | 6 [6 – 7] | 90 [90 – 90] | 16 [11 – 21] |
|  | 0.07 | 1088 [966 – 1258] | 3310 [2625 – 4353] | 6 [6 – 7] | 90 [90 – 90] | 23 [19 – 27] |

^1^ Outcomes are calculated from 400 runs with *scenario* ‘Heavy Rainfall’, *hygiene-level* 40%, and *frc-initial* to 0.5 mg/L.
