# Supplementary material for: Relative contributions of public and domestic transmission domains in cholera outbreaks in displacement camps: an exploratory agent-based modeling study
Source: Epidemiol Infect. 2026 May 13;154:e97. doi: 10.1017/S0950268826101575 (PMC13366369; doi:10.1017/S0950268826101575)
Supplement: Jaber et al. supplementary material [file S0950268826101575sup001.zip › Appendix_D.docx]

Relative contributions of public and domestic transmission domains in cholera outbreaks in displacement camps: an exploratory agent-based modelling study

Appendix D - Model calibration

We employed a Genetic Algorithm against one criterium, the number of bi-diurnal cholera infections over time during an outbreak in a displacement camp. The objective measure used was the sum of squared errors. We varied six parameters (Table 1) and performed a total of 4,800 model runs, including ten repetitions for each evaluated parameter set. The GA was run with settings *mutation-rate* = 0.5, *population-size* = 300, *crossover-rate* = 0.7, *tournament-size* = 3, *population-model* ‘steady-state-replace-worst’, and *chromosomeRepresentation* set to ‘MixedTypeChromosome’.

Table 1: Search space specification for the calibration of an agent-based model of cholera transmission.

| Parameter | Range | Increment |
| --- | --- | --- |
| hygiene-level | 0 - 80 | 1 |
| water-capacity | 0.2 - 0.8 | 0.01 |
| common-source^1^ | 6 - 8 | 1 |
| rewire-communities | 0.05 - 0.4 | 0.01 |
| rewire-blocks | 0 - 0.05 | 0.01 |
| exposure-probability | 0 - 1 | 0.01 |

^1^ *common-source* is the number of days (from the start of the simulation) with no transmission of *Vibrio cholerae* through water sources. It was only used for model calibration.
