# Supplementary material for: Relative contributions of public and domestic transmission domains in cholera outbreaks in displacement camps: an exploratory agent-based modeling study
Source: Epidemiol Infect. 2026 May 13;154:e97. doi: 10.1017/S0950268826101575 (PMC13366369; doi:10.1017/S0950268826101575)
Supplement: Jaber et al. supplementary material [file S0950268826101575sup001.zip › Appendix_A.docx]

Relative contributions of public and domestic transmission domains in cholera outbreaks in displacement camps: an exploratory agent-based modelling study

Appendix A - ODD protocol

The agent-based model (ABM) is implemented in NetLogo [1], a modelling environment for agent-based simulation. The model description follows the ODD (Overview, Design concepts, Details) protocol for describing individual- and agent-based models [2], as updated by Grimm et al. [3].

# Purpose and patterns

The overall purpose of our ABM is to describe cholera transmission dynamics in a stylized displacement camp. It aims to investigate how acute population influxes and extreme weather events may affect the percentage of infections emanating from both public and domestic transmission domains, as well as the size and timescale of cholera outbreaks. Ultimately, the ABM will be used to explore the effectiveness of cholera preparedness and response strategies in displacement camps.

The ABM was evaluated by its ability to reproduce two patterns: First, an epidemic curve characteristic of a past cholera outbreak in a displacement camp [4]. Second, an increased reliance on potentially contaminated water sources following an influx of new arrivals into a camp [5,6].

# Entities, state variables, and scales

Our ABM simulates cholera outbreaks in a stylized displacement camp. It includes the following entities: PoCs, shelters, water facilities, and health facilities. PoCs are persons of concern to the United Nations High Commissioner for Refugees (UNHCR). These include refugees, asylum-seekers, and internally displaced persons (IDPs). Camps are organized into modular planning units, among which are communities and blocks [7]. Based on UNHCR’s principles and standards for settlement planning [7], blocks in the ABM consist of 16 communities, composed of 16 shelters each. Shelters are collectives, i.e., aggregations of PoCs. They are characterized by their *shelter-location*, *water-quantity*, *stored-water-safe?*, *source-water-safe?*, and *stored-frc* (i.e., free residual chlorine [FRC]) among other state variables (Table 1). PoCs are assigned to shelters. They are endowed with a propensity to follow hygiene practices (e.g., handwashing with soap and water) and engage in activities as explained in [**Process overview and scheduling**](#_Process_overview_and). Their state variables include *health-status*, *activity-status*, *hygiene?*, and *water-level*. Water in camps is often chlorinated and delivered through tap stands [8]. In the ABM, each block includes a water facility entity, representing five water taps for 1250 people [8]. Water facilities are differentiated by their *water-facility-location*, *protected?*, *capacity*, *source-frc*, and *malfunction?* state variables. These deliver water throughout the day, with FRC set by a global parameter. Residual chlorine in treated water protects against post-collection microbiological contamination. However, once water is collected and stored in household-level containers, FRC decays over time [9]. There is one health facility in the camp, characterized by its *total-patients* (i.e., number of PoCs that were treated in the facility). State variables of the four entities included in our ABM and their descriptions are presented in Table 1 below.

Table 1: State variables characterizing entities in an agent-based model of cholera transmission developed on NetLogo.

| Entities | Variables | Description | Values | Type (Unit) |
| --- | --- | --- | --- | --- |
| PoCs | Activities | | | |
|  | *location* | Location of the PoC | shelter; water facility; health facility | NetLogo Agent |
|  | *household* | Shelter of the PoC | Shelter | NetLogo Agent |
|  | *activity-status* | Variable to record the activity of the PoC | none; water collection; visited; visiting; incapacitated; treatment | String |
|  | *maximum-contacts* | Maximum number of daily contacts the PoC can have | 0 – 12 | Integer (Contacts) |
|  | *visit-duration* | Time spent by the PoC in a relative’s shelter | 0 – 5 | Integer (Hours) |
|  | Water, Sanitation, and Hygiene Practices | | | |
|  | *water-level* | Water level of the PoC. Prompts the PoC to drink water when it is less than or equal to 0 | 0 – 0.9375 | Float (Litres) |
|  | *hygiene?* | Variable to specify whether the PoC follows proper hygiene practices (e.g., handwashing with soap) if there is sufficient water | true; false | Boolean |
|  | Health | | | |
|  | *health-status* | Health status of the PoC | susceptible; exposed; infected; recovered | String |
|  | *symptomatic?* | Variable to specify whether the PoC is symptomatic | true; false | Boolean |
|  | *gravis?* | Variable to specify whether the PoC has severe cholera (i.e., cholera gravis) | true; false | Boolean |
|  | *incubation-period* | Incubation period of cholera | 12 – 120 | Float (Hours) |
|  | *infectious-period* | Period in which the PoC sheds *Vibrio cholerae*, leading to the contamination of water storage containers | 24 – 432 | Float (Hours) |
| Shelters | Household Characteristics | | | |
|  | *shelter-location* | Patch where the shelter is located | min-pxcor -74; min-pycor -74; max-pxcor 75; max-pycor 75^*^ | NetLogo Agent |
|  | *shelter-block* | Block number of the shelter | 1 – 8 | Integer |
|  | *shelter-community* | Community number of the shelter | 1 – 16 | Integer |
|  | *household-size* | Number of PoCs belonging to the shelter | 4 – 6 | Integer (PoCs) |
|  | *household-members* | PoCs belonging to the shelter | PoCs | NetLogo Agentset |
|  | Water Quantity and Quality | | | |
|  | *stored-water-safe?* | Variable to record whether the water in the household storage container is safe to drink | true; false | Boolean |
|  | *source-water-safe?* | Variable to record whether the collected water was originally safe to drink | true; false | Boolean |
|  | *water-quantity* | Stored water quantity in the shelter | 0 – 20 | Float (Litres) |
|  | *stored-frc* | Residual chlorine in the household water storage container | 0 – *frc-initial* | Float (Milligrams per Litre) |
| Water Facilities | *water-facility-location* | Patch where the water facility is located | min-pxcor -74; min-pycor -74; max-pxcor 75; max-pycor 75 | NetLogo Agent |
|  | *protected?* | Variable to specify whether the water facility is protected | true; false | Boolean |
|  | *source-frc* | Residual chlorine at the water facility | 0 – *frc-initial* | Float (Milligrams per Litre) |
|  | *capacity* | Capacity of the water facility. A value of 1 corresponds to 1 tap per 250 people whereas 0.2 implies 1 functioning tap per 1250 people | 0.2 – 1 | Float |
|  | *malfunction?* | Variable to record whether the water facility is malfunctioning | true; false | Boolean |
| Health Facilities | *total-patients* | Number of PoCs being treated in the health facility | 0 – *health-capacity* | Integer (PoCs) |

^*^ min-pxcor, min-pycor, max-pxcor, and max-pycor are NetLogo reporters that determine the spatial extent of the virtual environment.

As for the spatial and temporal resolution and extent: the model is 150 x 150 patches, the environment representing 900000 m^2^. Every patch represents 40 m^2^ to allow for more than 5.5 m^2^ of covered living area per person [7], for a household of six. Every time step represents one hour to capture residual chlorine decay [9], and to allow for multiple water collection trips in a day. Simulations are run for a duration of 90 days or until outbreak termination, i.e., two consecutive weeks without new infections [10].

# Process overview and scheduling

Our ABM is meant to simulate a cholera outbreak starting from the introduction of *Vibrio cholerae* into the displacement camp until its termination, as defined in [**Entities, state variables, and scales**](#_Entities,_state_variables,). It includes 11 main processes. Three relate to shelters (*updateFRC*, *updateStoredWater*, *contaminateWater*), seven to PoCs (*updateHealthStatus, decideWaterCollection*, *collectWater*, *updateWaterLevels*, *drinkWater*, *decideVisits*, and *visit*), and one to water facilities (*damageWaterFacility*).

The simulation starts on 01-01-2024, at 00:00. Between 06:00 and 22:00 all 11 processes are executed every time step. At night (i.e., 22:00 onwards), only *updateFRC* and *updateHealthStatus* are executed. Here are the processes included in our ABM:

1. With *scenario* set to ‘Acute Population Influx’, water facilities execute *damageWaterFacility*, updating *malfunction?* and *capacity*. If all water facilities in the displacement camp are fully functional, one randomly selected water facility may malfunction for 48 hours (*capacity* is reduced by 0.2) with a probability of 0.009, equivalent to one malfunction event every week. This probability was informed by consultations with staff of the International Organization for Migration (IOM) Nigeria.
2. Shelters execute *updateFRC* and update their *stored-frc.* Further explanation is provided in [**Submodels**](#_Free_residual_chlorine).
3. PoCs execute *updateHealthStatus*, updating their *health-status* and the number of PoCs that were treated in the health facility (i.e., *total-patients*). Further explanation is provided in [**Submodels**](#_Epidemiology).
4. PoCs execute *decideWaterCollection*. One randomly selected and unoccupied PoC from each shelter may set *activity-status* to ‘water collection’. They choose among multiple water facilities in the displacement camp, as well as a polluted water source in the host community, and then modify their *location* to the selected water facility entity. Further explanation is provided in [**Submodels**](#_Water_collection).
5. PoCs whose *activity-status* is set to ‘water collection’ execute *collectWater* and remain at the water facility for a certain number of time steps before returning to their *household*. Once there, they update its *water-quantity*, *stored-water-safe?*, *source-water-safe?*, and *stored-frc*. Further explanation is provided in [**Submodels**](#_Water_collection).
6. Shelters execute *updateStoredWater* and reduce their *water-quantity* by 0.750 L/p/h, based on a consumption of 12 L/p/d for cooking and hygiene practices [8].
7. PoCs with *activity-status* other than ‘treatment’ execute *updateWaterLevels* and decrease their *water-level* by 0.1875 L/h, based on a consumption of 3 L/d for drinking and food [8].
8. Shelters execute *contaminateWater* and set *stored-water-safe?* to ‘false’ if *stored-frc* is less than 0.1 mg/L and any PoCs belonging to them are infected, symptomatic (i.e., mild, moderate, or severe cholera infections), and do not follow adequate hygiene practices. Asymptomatic infections shed much less *V. cholerae*, for a shorter amount of time [11]. We assume that these do not contribute to post-collection water contamination.
9. PoCs located in shelters execute *drinkWater*. They increase their *water-level* by a random number between 0.1875 L and 0.9375 L drawn from a uniform distribution. This amount is subtracted from *water-quantity* of the shelters they are in.
10. PoCs execute *decideVisit* and choose whether to visit friends and relatives, updating their *activity-status* to ‘visiting’. More specifically, unoccupied PoCs who have not yet exceeded their daily maximum allowed number of contacts (i.e., *maximum-contacts*) randomly choose a shelter among those of their relatives and modify their *location* to the shelter entity they selected. *maximum-contacts* is based on data from Digaale in Somaliland [12].
11. PoCs with *activity-status* set to ‘visiting’ execute *visit* and remain in the shelter they are visiting for a random duration between zero and five hours before returning to their *household*. This duration is based on data from Digaale in Somaliland [12].

Entities execute processes in a random order each time step to avoid artifacts due to execution order. ‘Observer’ processes take place at the end of every time step. These include incrementing the global variable *time* by one hour and updating the outputs on the NetLogo interface.

# Design concepts

## Basic principles

Both public and domestic transmission domains may play an important role in cholera outbreaks. However, the relative contributions of each of the two domains is context-specific and remains unclear.

Crooks and Hailegiorgis [13] developed an ABM of cholera spread within the Dadaab refugee camp in Kenya. They simulated outbreaks as emergent phenomena following either the contamination of a borehole or the spread of the pathogen through surface water run-off. Their work did not incorporate post-collection water contamination, nor did it explicitly distinguish between, and report on, the two transmission domains. Our ABM incorporates residual chlorine decay and post-collection water contamination, and explicitly distinguishes between public and domestic transmission domains. In the model, public domain transmission is conceptualized as occurring following exposure to *V. cholerae* through an environmental point source. In contrast, domestic domain transmission is conceived as taking place following the consumption of water contaminated at the household level by an infected and symptomatic PoC.

Cholera outbreaks in displacement camps tend to occur following shocks, including extreme weather events and acute population influxes [14–16]. The former may lead to the contamination of water sources. The latter overwhelms water, sanitation and hygiene facilities in camps, leading to a reliance on potentially contaminated water sources.

## Emergence

Exposure to *V. cholerae* is modelled as an emergent phenomenon following the interactions of camp inhabitants among themselves and with their surrounding environment. More specifically, it is a function of the PoCs’ choice among alternative water facility entities, *maximum-contacts* of PoCs, *hygiene?* among *household-members* of shelters, and the shelters’ social network.

## Adaptation

PoCs with *activity-status* set to ‘water collection’ chose between alternative water facility entities based on the time required to collect water, while holding a preference for water distributed inside the displacement camp (see [**Submodels**](#_Water_collection)). This is a direct objective seeking behaviour (i.e., one in which agents rank alternatives using a measure of how well each would meet some specific objective).

## Objectives

The objective measure used by PoCs to decide where to collect water from is water collection time. It is driven by the distance between the PoCs’ *household* and the water facility entities, as well as the number of PoCs with *location* set to each water facility. This measure is meant to capture a documented preference of camp inhabitants for shorter queues [6,17,18].

## Learning

Learning is not implemented in our ABM.

## Prediction

Our ABM incorporates implicit predictions (i.e., hidden or implied assumptions about the future consequences of decisions). PoCs assume that choosing the water facility entity associated with the lowest collection time will prevent their *household* running out of water.

## Sensing

PoCs sense their own *household*, *location*, *water-level*, *activity-status*, and *gravis?* state variables and the global variables *time* and *number-patients*. They know the maximum number of PoCs they are allowed to interact with in a day (i.e., *maximum-contacts*) and the *visit-duration*, as well as the number of PoCs at each water facility entity and their *capacity*. Finally, they sense *water-quantity* of their own and their relatives’ shelters. PoCs prefer water delivered inside the displacement camp, however, they cannot tell if a water facility entity is protected or not.

Shelters know each other’s *shelter-bloc* and *shelter-community*, the global variable *time*, and the parameter *frc-initial*. They also sense their own *stored-water-safe?*, *water-quantity*, and *stored-frc*, as well as their *household-members*’ *location*, *activity-status*, *health-status*, *symptomatic?*, and *hygiene?* state variables. Water facilities sense their *malfunction?* state variable, the global variable *time*, and parameters *frc-initial* and *water-capacity*.

## Interaction

Our ABM incorporates direct and indirect interactions. PoCs visit their unoccupied relatives and update the relatives’ *activity-status* from ‘none’ to ‘visited’. They consume water from their own *household* and their relatives’ shelters, reducing *water-quantity* there. PoCs also modify their *health-status* from ‘susceptible’ to ‘exposed’ through their interactions with shelter entities. Finally, all PoCs chose among the same set of water facility entities. Their choice affects *queuing-time* of other PoCs, and consequently, their water collection behaviour.

## Stochasticity

The ABM is initialized stochastically to ensure that each model run produces different results. This relates to where shelters and water facilities are located, the number of PoCs in each shelter, the index case, and *water-quantity* of shelters, as well as *hygiene?*, *water-level,* and *maximum-contacts* of PoCs. The social network linking shelters (and therefore, PoCs) is created through two rewiring probabilities (see [**Initialization**](#_Initialization)).

When *scenario* is set to ‘Heavy Rainfall’, *protected?* of one randomly selected water facility entity in the displacement camp is updated to ‘false’ and its *source-frc* is reduced to zero. Under *scenario* ‘Acute Population Influx’, half of water facilities in the displacement camp are randomly selected and their *capacity* is reduced. Probabilities are used to set *symptomatic?*, *gravis?*, *incubation-period*, *infectious-period*, and *visit-duration*, as well as to have PoCs die. Exposure to *V. cholerae* is also based on *exposure-probability* (see [**Submodels**](#_Epidemiology)). Water facility malfunction (i.e*., malfunction?*) is modelled as a stochastic process, with the probability of malfunction informed by a consultation with staff of IOM Nigeria.

## Collectives

Shelters are collectives in our ABM. Their state variables *water-quantity*, *stored-water-safe?, source-water-safe?*, and *stored-frc* are affected by PoCs. Simultaneously, they affect PoCs’ *health-status, water-level*, and water collection behaviour.

## Observation

The ‘Observer’ reports the cumulative number of infections attributed to each of the two transmission domains (excluding the index case) and the percentage of infections emanating from domestic transmission.

At the end of each day, the total numbers of susceptible, exposed, infected, and recovered PoCs are displayed in a plot. The daily number of new cholera cases (i.e., PoCs whose *health-status* was set to ‘infected’ that day) is also plotted. Lastly, the ABM allows users to visually explore spatial dynamics of cholera outbreaks by altering the colour of shelters with cholera infections among their *household-members*.

# Initialization

Our ABM represents a stylized displacement camp. Parameters reflect camps that follow UNHCR’s principles and standards for settlement planning [7], and the Sphere standards [8]. However, some state variables are based on data from specific displacement camps (e.g., Digaale in Somaliland [12]).

The *simulation-duration* is set to 90 days. The *outbreak-start-time* is set to the date and time ‘01-01-2024 00:00:00.000’. *Communities-per-block* and *shelters-per-community* are set to 16 [7], and the *number-of-shelters* is initialized to 2048 (Table 2).

Water facilities, equal to the number of blocks in the displacement camp, are initially created, at least 30 patches apart. Their *protected?* is set to ‘true’, *source-frc* to the user-defined *frc-initial* (between 0 and 1 mg/L), and *capacity* to the user-defined *water-capacity* (between 0.2 and 1). *malfunction?* is initially set to ‘false’. Shelters are then created around each water facility entity iteratively by *shelter-block* and *shelter-community* (i.e., block 1, community 1, followed by block 1 community 2… block 1 community 16, block 2 community 1, etc.). The *household-size* of shelters is set to a random integer between 4 and 6 [7]. *stored-water-safe?* and *source-water-safe?* are set to ‘true’, *water-quantity* is initialized to a random float between 4.5 L and 40 L drawn from a uniform distribution. An alternative water source (i.e., water facility entity located outside the displacement camp) is created with *protected?* set to ‘false’, *source-frc* to 0, and *capacity* to 1.

PoCs are assigned to the shelter where they were created (i.e., *household*). Their *location* is set to *household*. *hygiene?* is initialized through a random number generator to reach a percentage of PoCs following adequate hygiene practices equal to the user-defined *hygiene-level*. *health-status* is set to ‘susceptible’, *symptomatic?* and *gravis?* to ‘false’, *incubation-period* and *infectious-period* to 0. *activity-status* is initially set to ‘none’, and *water-level* is initialized to a random float between 0 L and 0.9375 L drawn from a uniform distribution. *maximum-contacts* are initialized based on data from Digaale in Somaliland [12]. Variables relevant to water collection are initialized based on *location* of PoCs, *shelter-location* of PoCs’ *household*, and *water-facility-location* of all water facility entities. These are expanded upon in [**Submodels**](#_Water_collection).

Under *scenario* ‘Acute Population Influx’, *hygiene?* of PoCs in the blocks housing new arrivals is reinitialized to reach *hygiene-level* - 20 (i.e., worsened hygiene practices among new arrivals), and *capacity* of the water facility entities in these blocks is reduced to 0.2 (i.e., one functioning tap per 1250 people). With *scenario* ‘Heavy Rainfall’, *protected?* of one randomly selected water facility in the displacement camp is set to ‘false’, and its *source-frc* is reduced to 0. The outbreak is initialized by prompting one PoC to set *health-status* to ‘exposed’, *symptomatic?* to ‘true’, and *gravis?* to ‘false’. The PoC then updates *incubation-period* and *infectious-period*. Further explanation is provided in [**Submodels**](#_Epidemiology).

PoCs primarily interact within communities and between communities of the same block. Interactions between blocks are less frequent. In our ABM, links are created between all shelters belonging to the same community. Then, with probabilities *rewire-communities* and *rewire-blocks* some links are severed, and new ones are formed between shelters belonging to different communities of the same block or to different blocks, respectively. These parameters were calibrated on data by Djeddah et al. [4].

Table 2: Parameters used for the initialization of an agent-based model of cholera transmission developed on NetLogo.

| Parameter | Description | Value | Source |
| --- | --- | --- | --- |
| Displacement Camp Characteristics | | | |
| shelters-per-community | Shelters in each community | 16 | United Nations High Commissioner for Refugees [7] |
| communities-per-block | Communities in each block | 16 | United Nations High Commissioner for Refugees [7] |
| number-of-shelters | Number of shelters | 2048, representing around 10000 camp inhabitants | Authors’ estimation to reach a camp population similar to that reported by Djeddah et al. [4] |
| health-capacity | Inpatient capacity in the health facility | 20 | Authors’ estimation for a population of 10000, assuming an attack rate for clinically apparent cholera of 5% and an average length of stay of 4 days [10] |
| frc-initial | Free residual chlorine at water facility entities in the displacement camp | Between 0 and 1 mg/L | Set on the NetLogo Interface |
| water-capacity | Capacity of water facility entities in the displacement camp | Between 0.2 and 1 | Set on the NetLogo Interface |
| hygiene-level | Percentage of camp inhabitants who follow adequate hygiene practices when provided with sufficient water | Between 0 and 100% | Set on the NetLogo Interface |
| scenario | Scenarios simulated through the agent-based model. These are detailed in the Experiments subsection | Displacement Camp, Acute Population Influx, Heavy Rainfall | Set on the NetLogo Interface |
| malfunction-probability | Probability that a water facility entity will experience a malfunction every time step while in operation in the Acute Population Influx *scenario* | 0.009 | Authors’ estimation based on consultations with the International Organization for Migration |
| malfunction-duration | Duration of malfunction | 48 hours | Authors’ estimation based on consultations with the International Organization for Migration |
| decrease-pp-hygiene | Percentage points decrease in *hygiene-level* in blocks housing new arrivals in the Acute Population Influx *scenario* | 20 | Authors’ assumption |
| capacity-blocks-arrivals | Capacity of water facility entities in blocks housing new arrivals in the Acute Population Influx *scenario* | 0.2 (i.e., one functioning tap per 1250 people) | Authors’ assumption |
| Social Network | | | |
| rewire-communities | Probability that a link between two shelters is severed and a new link is formed between shelters belonging to different communities within the same block | 0.25 | Calibration on data reported by Djeddah et al. [4] |
| rewire-blocks | Probability that a link between two shelters is severed and a new link is formed between shelters belonging to different blocks | 0.04 | Calibration on data reported by Djeddah et al. [4] |

# Input data

The model does not use input data to represent time-varying processes.

# Submodels

## Free residual chlorine decay

In line with the work of Ali et al. [9], residual chlorine decays every time step based on the integrated rate law, with $n=0.66$ and $k=0.1463$:

$$C={{(C_{0}}^{1-n}+\left( n-1 \right)kt)}^{\frac{1}{1-n}}$$

$$where C is the FRC in mg/L at time t$$

$$C_{0}is the initial FRC at time zero$$

$$n the dimensionless rate order$$

$$and k the rate constant with units mg^{1-n}L^{n-1}h^{-1}$$

Parameters $n$ and $k$ are based on data from refugee camps in South Sudan [9].

## Water collection

If *water-quantity* of shelters drops below the maximum amount their *household-members* may use in one time step, i.e., $\left( 0.9375+0.750 \right)\times n_{household members}$, one randomly selected, unoccupied PoC among *household-members* will set its *activity-status* to ‘collect water’. To choose among water facility entities, PoCs calculate the total collection time, based on the distance between their *household* and each water facility entity and the queues there.

PoCs’ *travel-time* represents the time required for one round trip to collect water. It is calculated for all water facility entities as follows: for each water facility entity $i$, the distance between a PoC’s *household* and water facility $i$, $d_{i}$, is converted to meters by multiplying it by 6.32 (one square patch represents 40 m^2^) (Table 3). Given that a distance of 1000 m is equivalent to a 30 min round trip [19], our *travel-time* (in min) is obtained by multiplying $d_{i}$ (in m) by 0.03:

$$t_{travel, i}= d_{i}\times6.32\times0.03$$

*queuing-time* is approximated based on a standard of three minutes to fill a 20 L container and a maximum supply capacity of five functioning taps:

$$t_{queuing,i}=n_{users}\times\frac{3}{5\times capacity}$$

$$t_{collection,i}=\frac{t_{travel,i}+t_{queuing,i}}{60}$$

For the alternative water facility, 45 min (equivalent to 1.5 km) are added to *travel-time*, and *queuing-time* is set to zero. PoCs select the water facility entity with the shortest *collection-time* (in hours) in the displacement camp, unless the time to collect water from the alternative water source is significantly shorter (see *alternative-source-threshold* in Table 3). This parameter is set to two, meant to incorporate a preference for water distributed inside the camp. Each round trip, PoCs refill their 20 L storage containers.

Table 3: Parameters relevant to water collection in an agent-based model of cholera transmission developed on NetLogo.

| Parameter | Description | Value | Source |
| --- | --- | --- | --- |
| dehydration-rate | Decrease in *water-level* each time step | 0.1875 | Authors’ estimation based on a daily water intake of 3 litres per person [8] |
| household-water-consumption-rate | Decrease in *water-quantity* each time step | 0.750 | Authors’ estimation based on a daily use of 12 litres per person for cooking and hygiene purposes [8] |
| walking-speed | Walking speed based on a round-trip time of 30 minutes over a distance of 1000 meters | 0.03 min/m | Cairncross [19] |
| time-fill-water-container | Time required to fill water container based on a flow rate of 7.5 L/min | 3 min | Authors’ estimation based on Sphere Association [8] |
| distance-between-two-patches | Distance from one patch to any of its four neighboring patches | 6.32 m | Authors’ estimation based on the United Nations High Commissioner for Refugees [7] |
| alternative-source-threshold | Ratio of the minimum collection time inside the displacement camp to that outside the camp, which must be exceeded to prompt camp inhabitants to collect water from the host community | 2 | Authors’ assumption |

## Epidemiology

PoCs update their *health-status* from ‘susceptible’ to ‘exposed’ if they drink water from an unprotected water facility entity with a probability *exposure-probability* calibrated on data by Djeddah et al. [4]. They set their *incubation-period* between 12 and 120 hours (Table 4), based on the distribution of cholera’s incubation period for O1 and O139 strains [20]. Afterwards, *health-status* updates to ‘infected’. Only 25% of infected PoCs are symptomatic, among which 8% are severe cholera cases (i.e., 2% of all infected PoCs) [21]. We assume that only symptomatic PoCs can contaminate water storage containers and contribute to domestic domain transmission. The *infectious-period* is then set, based on data published by Dizon [22].

Severe cholera cases seek to access the health facility entity (i.e., *activity-status* set to ‘treatment’). If unable to, their *activity-status* is set to ‘incapacitated’, and they remain inactive until they recover or die. This is determined by the number of available beds in the health facility. Once recovered, PoCs set their *health-status* to ‘recovered’. Those with severe cholera may die with probabilities of 1% and 50% depending on whether they receive treatment or not, respectively [11].

Table 4: Parameters relevant to cholera epidemiology in an agent-based model of cholera transmission developed on NetLogo.

| Parameter | Description | Value | Source |
| --- | --- | --- | --- |
| exposure-probability | Probability of collecting water contaminated with *Vibrio cholerae* from an unprotected and polluted water source | 0.35 | Calibration on data reported by Djeddah et al. [4] |
| incubation-period-data | Incubation period of *V. cholerae* | Between 12 and 120 hours | Azman et al. [20] |
| infectious-period-data | Period in which infected people shed *V. cholerae* | Between 24 and 432 hours | Dizon [22] |
| probability-death-no-treatment | Probability of death among severe cholera infections without treatment | 0.5 | Nelson et al. [11] |
| probability-death-with-treatment | Probability of death among severe cholera infections with adequate treatment | 0.01 | Nelson et al. [11] |
| probability-symptomatic | Probability that an infected person will develop symptomatic cholera (i.e., mild, moderate, or severe) | 0.25 | Gangarosa & Mosley [21] |
| probability-gravis | Probability that an infected person will develop severe cholera | 0.02 | Gangarosa & Mosley [21] |

References

1. **Wilensky U**. (1999) *NetLogo*. Evanston, IL: Center for Connected Learning and Computer-Based Modeling, Northwestern University.

2. **Grimm V, *et al.*** (2006) A standard protocol for describing individual-based and agent-based models. *Ecological Modelling*; **198**: 115–126. https://doi.org/10.1016/j.ecolmodel.2006.04.023

3. **Grimm V, *et al.*** (2020) The ODD protocol for describing agent-based and other simulation models: a second update to improve clarity, replication, and structural realism. *Journal of Artificial Societies and Social Simulation*; **23**: 7. https://doi.org/10.18564/jasss.4259

4. **Djeddah C, *et al.*** (1988) An outbreak of cholera in a refugee camp in Africa. *European Journal of Epidemiology*; **4**: 227–230. https://doi.org/10.1007/BF00144757

5. **Kisera N, *et al.*** (2020) A descriptive cross-sectional study of cholera at Kakuma and Kalobeyei refugee camps, Kenya in 2018. *Pan African Medical Journal*; **37**. https://doi.org/10.11604/pamj.2020.37.197.24798

6. **Swerdlow DL, *et al.*** (1997) Epidemic cholera among refugees in Malawi, Africa: treatment and transmission. *Epidemiology and Infection*; **118**: 207–214. https://doi.org/10.1017/s0950268896007352

7. **United Nations High Commissioner for Refugees**. (2024) *Principles & standards for settlement planning*. https://emergency.unhcr.org/emergency-assistance/shelter-camp-and-settlement/formal-settlements/principles-standards-settlement-planning [last accessed on 19 June 2024]

8. **Sphere Association**. (2018) *The Sphere handbook: humanitarian charter and minimum standards in humanitarian response*. Fourth edition. Geneva, Switzerland. https://spherestandards.org/wp-content/uploads/Sphere-Handbook-2018-EN.pdf [last accessed on 23 June 2024]

9. **Ali SI, Ali SS, Fesselet J-F**. (2021) Evidence-based chlorination targets for household water safety in humanitarian settings: recommendations from a multi-site study in refugee camps in South Sudan, Jordan, and Rwanda. *Water Research*; **189**: 116642. https://doi.org/10.1016/j.watres.2020.116642

10. **Olson D, Fesselet J-F, Grouzard V.** (2018) *Management of a Cholera Epidemic*. Médecins Sans Frontières. https://medicalguidelines.msf.org/sites/default/files/pdf/guideline-800-en.pdf (accessed 19 June 2024)

11. **Nelson EJ, *et al.*** (2009) Cholera transmission: the host, pathogen and bacteriophage dynamic. *Nature Reviews Microbiology*; **7**: 693–702. https://doi.org/10.1038/nrmicro2204

12. **van Zandvoort K, *et al.*** (2022) Social contacts and other risk factors for respiratory infections among internally displaced people in Somaliland. *Epidemics*; **41**: 100625. https://doi.org/10.1016/j.epidem.2022.100625

13. **Crooks AT, Hailegiorgis AB**. (2014) An agent-based modeling approach applied to the spread of cholera. *Environmental Modelling & Software*; **62**: 164–177. https://doi.org/10.1016/j.envsoft.2014.08.027

14. **Burnet E, Rudge JW**. (2019) Diarrhoeal disease outbreaks associated with sanitation provision failures in refugee camps worldwide: a literature review. *Waterlines*; **38**: 36–63. https://doi.org/10.3362/1756-3488.18-00018

15. **Shannon K, *et al.*** (2019) Cholera prevention and control in refugee settings: successes and continued challenges. *PLOS Neglected Tropical Diseases*; **13**: e0007347. https://doi.org/10.1371/journal.pntd.0007347

16. **Jaber T, *et al.*** (2024) Outbreaks of faecal-orally transmitted diseases in displacement camps: a scoping review of pathogens, risk factors, exposure routes, and drivers of transmission. *Global Public Health*; **19**: 2380847. https://doi.org/10.1080/17441692.2024.2380847

17. **Monje F, *et al.*** (2020) A prolonged cholera outbreak caused by drinking contaminated stream water, Kyangwali refugee settlement, Hoima District, Western Uganda: 2018. *Infectious Diseases of Poverty*; **9**: 154. https://doi.org/10.1186/s40249-020-00761-9

18. **Mulholland K**. (1985) Cholera in Sudan: an account of an epidemic in a refugee camp in eastern Sudan, May–June 1985. *Disasters*; **9**: 247–258. https://doi.org/10.1111/j.1467-7717.1985.tb00947.x

19. **Cairncross S**. (1988) Domestic water supply in rural Africa. In: Rimmer D, ed. *Rural Transformation in Tropical Africa*. London, UK: Belhaven Press, pp. 46–63.

20. **Azman AS, *et al.*** (2013) The incubation period of cholera: a systematic review. *Journal of Infection*; **66**: 432–438. https://doi.org/10.1016/j.jinf.2012.11.013

21. **Gangarosa EJ, Mosley WH**. (1974) Epidemiology and surveillance of cholera. In: Barua D, Burrows W, eds. *Cholera*. Philadelphia: W. B. Saunders Co., pp. 381–403.

22. **Dizon JJ**. (1974) Cholera carriers. In: Barua D, Burrows W, eds. *Cholera*. Philadelphia: W. B. Saunders Co., pp. 367–379.
